# Traditional knowledge of animal-derived medicines used by Gelao community in Northern Guizhou, China

**DOI:** 10.1186/s13002-024-00669-w

**Published:** 2024-03-01

**Authors:** Xiaoqi Liu, Shuo Li, Yi Feng, Xingxing Chen, Yuhan Ma, Hai Xiao, Yongxia Zhao, Sha Liu, Guishen Zheng, Xiujuan Yang, Faming Wu, Jian Xie

**Affiliations:** 1 School of Pharmacy, Zunyi Medical University, Zunyi, 563000 China; 2 School of Pharmacy, Gansu University of Chinese Medicine, Lanzhou, 730101 Gansu China; 3 Maternal and Child Health Care Hospital, Zunyi, 563000 China

**Keywords:** Ethnobiology, Gelao ethnicity, Traditional medicine, Animal-derived medicines, Sustainable utilization

## Abstract

**Introduction:**

This study aims to document and preserve the traditional medicinal knowledge of the Gelao community in Northern Guizhou, China, providing valuable insights for modern pharmacological research and the development of these traditional remedies.

**Methods:**

Our methodology encompassed a blend of literature review, community interviews, and participatory observation to delve into the traditional knowledge of animal-derived medicines among the Gelao community. We employed quantitative ethnological and ecological assessment techniques to evaluate the significance of these practices. Informed consent was secured before conducting interviews, with a focus on ascertaining the types of medicines familiar to the informants, including their local names, sources, methods of preparation, application techniques, diseases treated, frequency of use, and safety considerations.

**Results:**

Our research cataloged 55 varieties of animal-derived medicines utilized by the Gelao people. Out of these, 34 originate from wild animals, mainly encompassing small insects, reptiles, and aquatic species; the remaining 21 are derived from domesticated animals, largely involving their tissues, organs, and various physiological or pathological by-products. These medicines are primarily applied in treating pediatric ailments (13 types), internal disorders (11 types), gynecological issues (3 types), dermatological problems (7 types), ENT conditions (3 types), trauma-related injuries (5 types), joint and bone ailments (5 types), infections (2 types), dental issues (2 types), and urolithiasis (1 type), with three types being used for other miscellaneous conditions. Commonly utilized medicines, such as honey, Blaps beetle, chicken gallstones, and snake-based products, are preferred for their availability, edibility, and safety within the Gelao communities.

**Conclusion:**

The Gelao community’s traditional medicines represent a rich diversity of animal sources, showcasing extensive expertise and knowledge in their processing and clinical applications. This wealth of traditional knowledge offers novel perspectives for the contemporary pharmacological study and development of these remedies. Additionally, our research plays a crucial role in aiding the preservation and continuation of this invaluable cultural heritage.

## Background

Animal-derived medicines, substances extracted from animals or their by-products with therapeutic or disease prevention properties, are integral to human medicinal culture [1]. Their discovery is intricately linked to human activities like hunting and livestock rearing. Initially, humans consumed raw animal products, including meat and blood. However, the advent of controlled fire use marked a significant shift in our interaction with animal-based resources. This breakthrough led to a broader exposure to various animal components, such as meat, fat, organs, bones, and marrow, and a deeper understanding of their nutritional benefits, potential toxicities, and medicinal qualities. These medicines substances are characterized by their diversity, encompassing both vertebrates (mammals, birds, reptiles, amphibians, fish) and invertebrates (arthropods, mollusks, echinoderms, coelenterates, sponges) [2]. These medicines can be further classified based on the animal part used, into ‘whole-body’ and local. ‘whole-body’ refers to using the entire animal or its major parts as medicine, like centipedes, scorpions, beehives, etc.; whereas local refers to using specific parts or organs of an animal, like deer antlers, fish gall, snake bile, etc. [3]. Based on the nature of these medicines, they can be divided into unprocessed and processed categories. Unprocessed medicines are those used directly or with minimal treatment, such as honey, royal jelly, propolis, etc., whereas processed medicines require methods like drying, calcination, and processing, such as cow bezoar, musk, deer antler glue, etc. [4].

The historical application of animal-derived medicines traces back to primitive societies, where components derived from animals were employed for wound healing, stopping bleeding, and alleviating pain [5]. As humans gained deeper understanding of nature, they also identified animals or animal parts with special effects, such as snakes, centipedes, scorpions, bees, silkworms, and deliberately used them as medicines [6]. China, one of the earliest countries to embrace natural medicines [7], has a documented history of such practices that spans over three thousand years [8]. Presently, traditional Chinese medicines include over ten thousand species of plants, animals, and minerals [9]. Significantly, China’s diverse and unified nation is home to 55 ethnic minorities besides the Han nationality, each with its unique medicinal culture. The interaction among these different ethnicities has contributed to the continuous evolution of their distinctive ethnic medicinal cultures, culminating in a complex and rich medicinal cultural system [10]. In these ethnic medicine systems, while plant-based medicines are predominant, animal-derived medicines, though less in number, are irreplaceable in various ethnic healthcare practices. For instance, in Chinese ethnic minorities, the Yi use musk for snake venom treatment, the Naxi utilize grasshoppers for blood extraction, and the Oroqen use deer heart blood mixed with brown sugar and yellow wine for treating rapid heartbeat [11].

During the Spring and Autumn and Warring States periods, the ‘Classic of Mountains and Seas’ documented 67 types of animal-derived medicines [12]. This ancient text marked the beginning of a long tradition of recording animal medicines in Chinese literature. Subsequently, the ‘Shennong Bencao Jing’ from the Qin and Han dynasties detailed 65 types of animal medicines [13], including well-known substances like deer antler, musk, and cow bezoar, which remain integral to modern medicinal practices. The ‘Newly Revised Materia Medica’ compiled in the Tang dynasty further expanded this list to 128 types of animal medicines [14]. In the Ming dynasty, Li Shizhen’s seminal work, the ‘Compendium of Materia Medica,’ included an extensive compilation of 461 types of animal medicines. This comprehensive text categorized these medicines into various sections based on their source—insects, scales, shells, birds, beasts, and even humans [15]. The ‘Supplement to the Compendium of Materia Medica’ by Zhao Xuemin during the Qing dynasty listed 128 types of animal medicines [16]. The modern ‘Encyclopedia of Traditional Chinese Medicine’ reflects the continuous growth in this field, listing as many as 740 types of animal medicines [17]. According to the results of the third national survey of medicinal resources in China, medicinal animals encompass 11 phyla, 414 families, 879 genera, and 1581 species, including mammals, birds, reptiles, amphibians, fishes [18].

Animal-derived medicines hold a significant place among various ethnic groups within China. For example, the Tibetan medical text ‘Jingzhu Materia Medica’ dedicates an entire chapter to animal-derived medicines [19], elaborating on a wide range of such medicines, their compound formulations, and processing methods. Similarly, the ‘Standard of Mongolian Medicine,’ a pivotal text in Mongolian medical literature, documents a substantial number of animal-derived medicines, providing detailed descriptions of their morphology, parts, collection timing, processing methods, properties, functions, indications, and applications [20]. Other Chinese ethnic groups, including the Uyghur, Zhuang, Shui, and Miao, also possess rich traditions and extensive written records of using animal-derived medicines. Chinese scholars have made significant contributions to treating complex diseases with these medicines. Notable examples include the use of leech extract for thrombotic diseases [21], scorpion for cardiovascular diseases [22], and centipede for osteomyelitis [23]. These studies underscore the unique therapeutic effects of animal-derived medicines in treating certain ailments, offering benefits that plant medicines may not provide. With the advancements in science and research, the development and application of modern pharmaceuticals have underscored the value of traditional animal-derived medicines. The extensive and varied use of medicinal animals for disease prevention and treatment in China is unparalleled globally and represents a significant contribution to the world’s medicinal heritage.

Globally, the employment of animal-derived medicinal substances illustrates the profound and enduring relationship between humanity and the natural world [24–27]. The World Health Organization (WHO) reports that 11.1% of 252 essential chemicals originate from plants, and 8.7% from animals. In the USA, 27 out of 150 currently used prescription drugs are derived from animals. Approximately 15–20% of ethnic medicines in India have an animal origin. The Unani system of medicine records nearly 200 animal-based drugs. In Pakistan, 31 medicines (9% of all medicines) are noted in folk medicine catalogs. In Brazil, a diverse array of medicinal substances sourced from mammals, birds, amphibians, and fishes are employed to combat parasitic infections, and to treat diseases affecting the respiratory, endocrine, and oncological systems [28–30]. A detailed ethnobiological survey in the Gran Chaco region of Argentina has documented 199 medicinal uses across 72 species distributed among 52 families, showcasing the rich animal pharmacopoeia integral to local medicinal practices [31]. Owing to the active components of medicinal animals having strong effects, small dosages, significant and specific therapeutic effects, and low toxicity, coupled with their wide range of sources and extensive experiences in gathering and using these medicines among the public, it is anticipated that with the continuous development of science and technology, medicinal animals will have a broad future in disease prevention and treatment. According to the World Health Organization (WHO), after extensive consultation with experts worldwide, the twenty-first century is predicted to be the century of animal medicine research. The study and application of medicinal animals are expected to further develop.

The Gelao people, an ethnic minority native to the southwestern regions of China, have traditionally inhabited the challenging and remote terrains of northern Guizhou [32]. This has necessitated their reliance on the diverse resources readily available in their vicinity for disease management. Compared to the abundant and diverse local plant-based medicinal resources, the number of animal-derived medicines is relatively lesser. However, these animal-derived medicines have played a crucial role in the healthcare practices of the Gelao community and continues to be a cornerstone of their medicinal traditions [33]. Inhabiting areas characterized by fluctuating environmental conditions and limited resources, the Gelao community have honed the art of survival, transforming their natural surroundings into a repository of medicinal knowledge passed down through generations. Yet, there exists a notable gap in systematic research concerning the Gelao’s traditional knowledge in utilizing animal-derived medicines. To date, only a few records of such medicines have been gathered in texts like the ‘Traditional Medicinal Atlas of the Gelao Community’ and ‘Gelao Medicine’ [34]. A more pressing concern is the faster rate of disappearance of the traditional knowledge of using animal-derived medicines compared to that of plant-based herbal medicines. Thus, Preserving and passing down this traditional knowledge, which encapsulates the wisdom of countless generations, is of utmost importance.

This study seeks to meticulously document and safeguard the indigenous medicinal knowledge of the Gelao community in Northern Guizhou, China. It aims to delve into the cultural underpinnings, distinctive characteristics, and utilization patterns of these medicinal practices within the Gelao society. Furthermore, this study is dedicated to assessing the importance and prospective applications of the Gelao community’s traditional knowledge concerning animal-derived medicines in the realm of modern healthcare. By providing deep insights into the amalgamation of these practices with their extensive ethnomedicinal and cultural heritage, our research endeavors to make a significant contribution to the global comprehension and valuation of ethnobiological and ethnomedicinal diversity.

## Materials and methods

### Research area

**Fig. 1:**
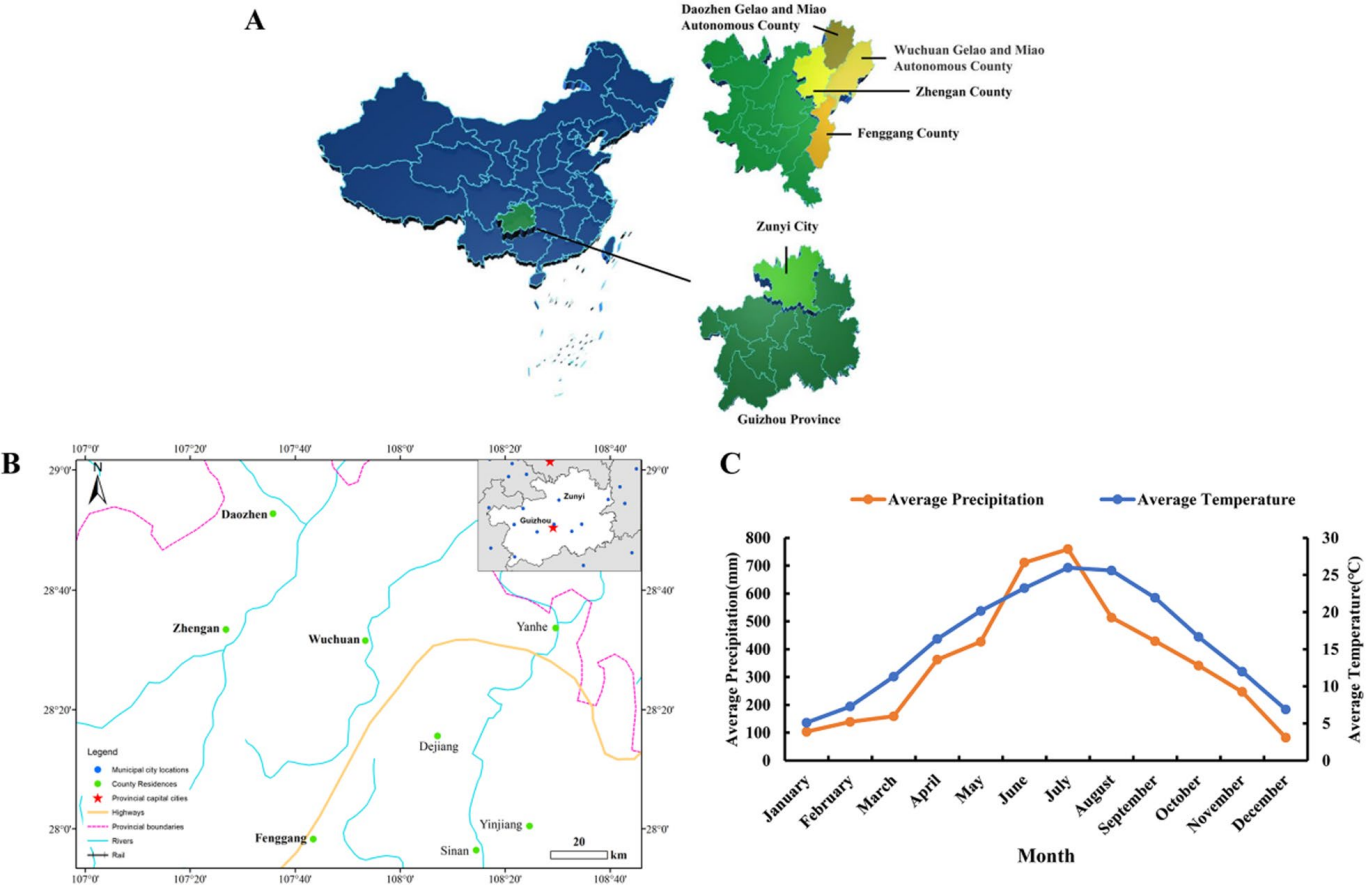
Research Area. **A** Geographical Scope, Daozhen Gelao-Miao autonomous county, Wuchuan Gelao-Miao autonomous county, Zhengan county and Fenggang County in Zunyi City, Guizhou Province. **B** The transportation network within the study area. **C** Climatic Conditions within the study area

This study was conducted in the northern part of Guizhou Province, China, specifically within the jurisdiction of Zunyi City, encompassing the counties of Daozhen, Wuchuan, Zhengan, and Fenggang (Fig. 1). The focus of the study is the Gelao community, an ethnic minority residing in this region. The Gelao, part of the Sino-Tibetan language family, uniquely communicate using the Chinese script, as they do not have their own written language. Their traditional cultural beliefs include the worship of the Bamboo King, the Barbarian King Ancestor, and the Mountain God. As per the ‘China Statistical Yearbook-2021’ [35], the Gelao population in China is estimated at approximately 677,000, with about 70% of this population living within our study area [36].

The research area is distinguished by its typical karst topography, featuring diverse landscapes such as high mountains, hills, basins, and terraces. The elevation range is notable, from the highest point at 1939 m to the lowest at 317 m, with an average elevation of around 1200 m. This region experiences a mid-subtropical humid monsoonal climate, characterized by mild weather, distinct seasonal changes, and substantial rainfall. The average annual rainfall is 1076 mm, complemented by a frost-free period averaging 290 days and an average annual temperature of approximately 16.14 °C. In terms of vegetation, the area above 1400 m is predominantly covered with deciduous broadleaf forests, bamboo groves, and shrubbery. Below this elevation, the landscape is a mix of natural and cultivated vegetation, with a forest coverage rate exceeding 60%. This rich and varied vegetation is crucial for the local ecosystem and biodiversity.

The region also includes the national-level Dashahai Nature Reserve. A recent 2022 survey revealed that this reserve hosts a remarkable variety of wildlife: 543 families, 2270 genera, and 5356 species (and varieties) of wildlife, including 67 nationally protected and 86 Guizhou-protected species. Among these, there are 54 orders, 282 families, and 1309 genera of 2002 species of wildlife. Vertebrates include 33 orders, 88 families, 240 genera of 337 species, comprising 57 species of mammals (across 8 orders, 21 families, 48 genera), 174 species of birds (15 orders, 38 families, 111 genera), 27 species of amphibians (2 orders, 8 families, 15 genera), 34 species of reptiles (3 orders, 10 families, 28 genera, including subspecies), and 45 species and subgenera of fish (5 orders, 11 families, 38 genera). Insects (including arachnids and pre-orders) consist of 21 orders, 194 families, 1069 genera of 1665 species and subspecies [37]. The reserve features 36 nationally protected rare animals [38]. These natural conditions offer the Gelao community an abundant and diverse array of animal-derived medicines. Their utilization of such resources reflects a deep-seated adaptation to and exploitation of the natural environment, developed through long-standing practical experience in their production and daily life.

## Collection of ethnobiological information

In this study, a blend of multiple research methods was employed to garner a comprehensive understanding of the Gelao community’s traditional knowledge regarding the use of animal-derived medicines. These methods included fieldwork, telephonic surveys, and literature reviews, each contributing uniquely to the depth and breadth of the data collected. Fieldwork, being the cornerstone of this research, was considered the most direct and effective approach. It involved a range of techniques: Key informant interviews were structured around the core ‘5W + 1H’ questions (When, Where, Who, Why, What, How) [39, 40]. This method proved pivotal in collecting traditional knowledge from Gelao doctors about animal-derived medicines. The interviews provided insights into the informants’ backgrounds, local names of animals and their medicinal parts, processing methods, usage, efficacy, and safety details. Semi-structured interviews conducted primarily at traditional medicine stalls in temporary markets, these interviews involved filling out questionnaires. This approach was efficient for gathering a large volume of information quickly. By comparing and verifying similar medicinal materials, the accuracy and reliability of the data were enhanced. We applied participatory observation [41, 42], which included following Gelao doctors and local residents (guides) to obtain specimens of traditional animal-derived medicines and learn the skills of processing, concocting, and compounding these medicines. This in-depth participation not only helped in collecting physical evidence but also in understanding the practical application and practices of traditional knowledge. This approach provided a comprehensive understanding of the importance and complexity of animal-derived medicines in Gelao culture.

### Quantitative evaluation

The diversity of medicinal information gathered from various villages was evaluated using the Simpson Index (*D*), defined as *D* = ∑Pi^2^. Here, ‘*D*’ represents the diversity index, and ‘Pi’ denotes the proportion of informants who reported on medicine ‘*i*’ in relation to all medicines [43].

The richness of medicinal information across villages was quantified using the Shannon–Wiener Index (*H*′). This index is calculated as *H*′ = − ∑Pi∙ln(Pi), where ‘Pi’ is the likelihood of the initial informant in a village mentioning medicine ‘*i*.’ Here, Pi = Ni/*N*, with ‘Ni’ being the count of informants reporting on medicine ‘*i*’ in the village, and ‘*N*’ representing the total number of informants in that village [44].

The similarity in medicinal information between various villages was gauged using the Sorenson Index (Cs), calculated as Cs = 2*j*/(*a* + *b*). Here, ‘*j*’ signifies the count of medicinal species common to villages A and B, ‘*a*’ is the total number of medicinal species in village A, and ‘*b*’ represents the total number in village B [45, 46].

The Utilization Frequency (HUF) was employed to assess local adaptation strategies and the extent of medicinal resource utilization. It is defined as *f* = Nm/Ni, where ‘*f*’ stands for Utilization Frequency, ‘Nm’ is the number of informants who mentioned the medicine, and ‘Ni’ denotes the total count of informants [47].

The National Cultural Significance Index (NCSI) was utilized to gauge the significance of each medicine in local life. NCSI is calculated as NCSI = FQI × AI × FUI × PUI × MFI × CEI × DSI × 10^–2^. Here, ‘FQI’ represents the Frequency of Quotient Index (the ratio of informants mentioning a specific medicine to all informants), ‘AI’ is the Availability Index, ‘FUI’ denotes the Frequency of Utilization Index, ‘PUI’ is the Parts used index, ‘MFI’ stands for the Multifunctional use Index, ‘CEI’ is the Curative Effect Index, and ‘DSI’ refers to the Drug Safety Index. These indices were established and graded following the ‘Methodology of Ethnobotany’ [43], with appropriate values assigned [48].

### Specimen identification

The animal sources of the collected medicinal materials were identified utilizing resources such as the ‘Colored Atlas of Chinese Medicinal Animals’ [49] and ‘Important Chinese Medicinal Insects’ [50]. Voucher specimens, specifically bottle specimens, were prepared. All gathered data were systematically organized, analyzed in line with the research objectives, and depicted in various charts and tables. These voucher specimens are preserved at the Traditional Chinese Medicine Specimen Museum of the Medical College, Zunyi Medical University.

## Results

### Characteristics of informants

This study was carried out in 10 Gelao villages across northern Guizhou, gathering data from 50 credible informants. These individuals were adept in providing insights into the usage of animal-derived medicines among the Gelao, encompassing both herbal doctors and medicinal material traders, averaging about 5 informants per village. All 50 participants had firsthand experience with Gelao traditional therapies. Informed consent was secured from each participant prior to the interviews, and they duly signed the consent forms. The survey spanned across 4 villages each in Daozhen and Wuchuan Counties, and 2 villages each in Zhengan and Fenggang Counties (refer to Table 1).

**Table 1:** Basic information of study areas

County	Location	Altitude	Climate	Population	Main ethnic	Main language	GDP/person	Investigation site	Longitude and latitude	Number of participants	Gender ratio	Occupation
Daozhen	North of Guizhou Province (E107° 21′–107° 51′; N28° 36′–29° 13′)	317.9–1939.9 m	Subtropical humid monsoon climate	240,000	Glao (48%)/Han/Miao/Tujia	Chinese/Miao/Gelao	¥34,000	Sanlong village, Longxing town, Daoxian county	E107° 22′; N28° 42′	8	Male: Female = 5:3	Farmers, rural doctors
								Luolong village, Longxing town, Daoxian county	E107° 41′; N29° 3′	4	Male: Female = 1:1	Farmers, government staff
								Zhaoshan village, Zhongping town, Daoxian county	E107° 41′; N28° 41′	3	Male: Female = 2:1	farmers
Wuchuan	North of Guizhou Province E107° 30′–108° 13′; N28° 11′–29° 05′	325.3–1743 m	Subtropical plateau humid monsoon climate	480,000	Glao (44%)/Han/Miao	Chinese/Miao/Gelao	¥360,00	Shankeng village, Daping town, Wuchuan county	E108° 2′; N28° 37′	7	Male: Female = 4:3	Farmers, rural doctors
								Tongxin village, Maotian town, Wuchuan county	E108° 5′; N28° 54′	5	Male: Female = 4:1	Farmers
								Shanshui village, Duluo town, Wuchuan county	E107° 53'; N28° 29'	3	Male: Female = 1:2	Farmers
Zhengan	North of Guizhou Province E107° 4′–107° 41′; N28° 9′–28° 51′	448–1838 m	Subtropical humid monsoon climate	660,000	Han(65%)/Glao/Miao	Chinese/Miao/Gelao	¥350,00	Guangda village, Gelin town, Zhengan county	E107° 30′; N28° 37′	6	Male: Female = 2:1	Farmers、teacher
								Shiyin village, Miliang town, Zhengan county	E107° 25′; N28° 23′	4	Male: Female = 1:3	Farmers, government staff
Fenggang	North of Guizhou Province E107° 31′–107° 56′; N27° 32′–28° 21′	398–1406.1 m	Subtropical humid monsoon climate	448,000	Han(80%)/Glao/Miao	Chinese/Miao/Gelao	¥270,00	Pingtouxi village, Tianqiao town, Fenggang county	E107° 52′; N27° 39′	5	Male: Female = 2:3	Farmers
								Suiyang village, Yantai town, Fenggang county	E107° 47′; N28° 10′	5	Male: Female = 2:3	Farmers, merchants

The survey revealed that female informants could recognize an average of 17 different animal-derived medicines, whereas male informants identified around 28 on average. This finding starkly contrasts with our earlier survey conducted in the multi-ethnic areas at the junction of Gansu, Ningxia, and Inner Mongolia [51]. In Gelao societies, traditional doctors are predominantly male. However, female informants play a vital role in supporting these traditional doctors in disease treatment and also as recipients of medicinal knowledge.

**Fig. 2:**
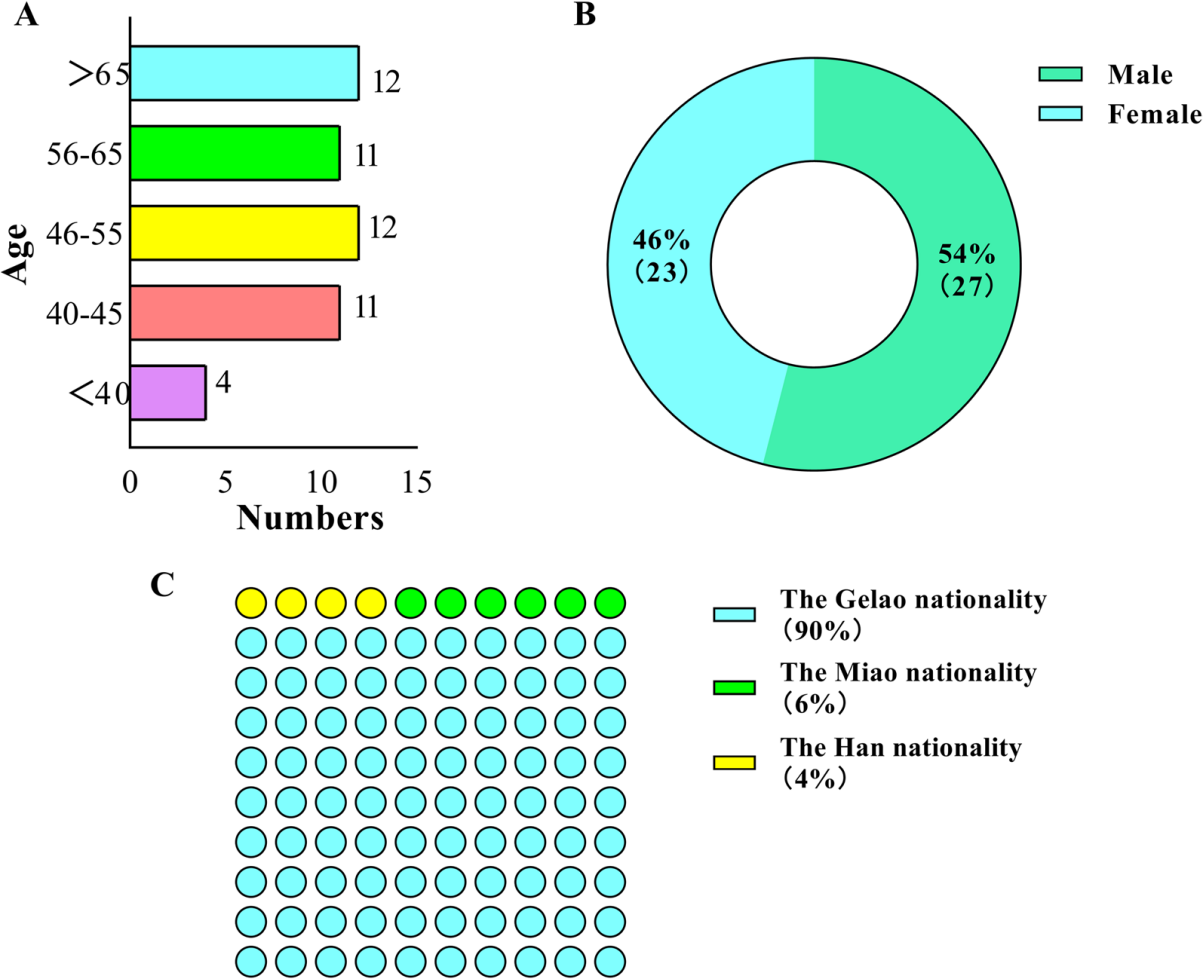
Demographic profile of informants. **A** Age structure. **B** Gender ratio. **C** Ethnicity ratio

Shows their ages varied from 34 to 89 years. The age distribution was as follows: 4 informants were under 40, 11 were aged 40–45, 12 between 46 and 55, another 11 between 56 and 65, and 12 were above 65 years of age. The gender breakdown included 27 males and 23 females, yielding a male-to-female ratio of 1.17:1. Of these informants, 45 were from the Gelao ethnic group, comprising 90% of the total, along with 3 Miao and 2 Han informants. The study also found that older informants possessed a deeper understanding of traditional medicines. While some knowledge of traditional animal-derived medicines was acquired from informants under 45, those actively involved in traditional Gelao medical practices were typically over 55. Also, these Gelao doctors generally had a lower level of formal education, mostly not exceeding high school, and with increasing age, their experience became richer, but their educational level remained relatively low (Fig. 2).

### Animal-derived medicines used by the Gelao community

**Fig. 3:**
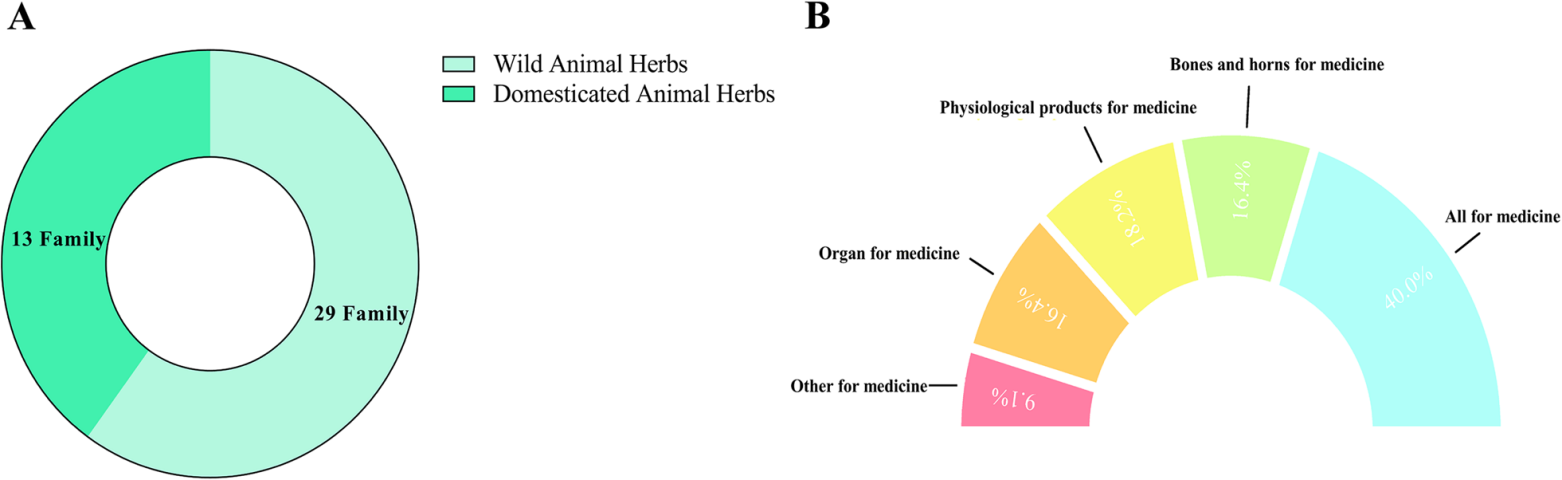
Gelao community’s use of animal-derived medicines. **A** Types of medicines. **B** Methods of medicine application

Our study identified a total of 55 varieties of animal-derived medicines used by the Gelao community, detailed in Table 2. This includes 33 types of wild animal-based substances, covering 29 distinct families, and 22 varieties derived from domestic animals, belonging to 13 different families (Fig. 3A). The categorization of these medicines is as follows: 22 types utilizing the entire animal, mostly small insects (e.g., Dinidoridae (Stål, 1867), *Lytta vesicatoria* (Linnaeus, 1758), Carabidae (Latreille, 1802)), mollusks (e.g., Lumbricina), arthropods (e.g., *Scolopendra subspinipes* (Leach, 1815)), and aquatic animals (e.g., *Muraena alba* (Zuiew, 1793), *Andrias davidianus* (Blanchard, 1871)). Typically, these are either dried or cooked whole before medicinal use, or combined with other substances, and are known for their efficacy in dispelling wind, alleviating pain and swelling, and detoxifying and killing insects. Nine types involve the use of organs (e.g., *Dendroaspis polylepis* (Günther, 1864), Ursus thibetanus (G. Baron Cuvier, 1823), *Gallus gallus*, *Sus scrofa* (Linnaeus, 1758)), typically processed after extraction for clearing heat, detoxification, benefiting gallbladder and diuresis, and improving vision. Nine types include the use of bones and horns (e.g., *Cervus canadensis* (Erxleben, 1777*)*, *Capra hircus* (Linnaeus, 1758), *Sus scrofa* (Linnaeus, 1758)), usually ground into powder or slices for strengthening qi and blood, fortifying muscles and bones, and nourishing yin and moistening dryness. Ten types involve physiological products (e.g., *Bombyx mori* (Linnaeus, 1758), Bos taurus (Linnaeus, 1758)), typically collected or extracted for clearing heat, resolving phlegm, stopping coughs, and nourishing and beautifying. Five other types are also mentioned (Fig. 3B).

**Table 2:** Animal-derived medicines used by the Gelao people

Local name	Name of Chinese medicine	Species	Family	Used part	Processing method	Main treatment	Mainly used in conjunction with medicines	Usage	Voucher numbers
Xiao Feng Er	Silkworm Chrysalis	*Bombyx morio* (Linnaeus, 1767)	Bombycidae	All	Drying	cure noma of children	Silkworm chrysalis, honey	Stir-fried for consumption	ZY-2023-W-003
Jiang Can	White Stiffy Silkworm	*Bombyx morio* (Linnaeus, 1767)	Bombycidae	Whole	Low temperature drying	Coughing	Good end tea, white silkworms	Ground and taken internally	ZY-2023-Z-002
Can Shi	Silkworm sand	*Bombyx morio* (Linnaeus, 1767)	Bombycidae	Physiological products	Drying	Treatment of hemorrhagic gonorrhea	Silkworm Sand	Ground and taken with wine	ZY-2023-Z-013
Zhi Liao Pi	Cicada Shedding	Cicadidae (Latreille, 1802)	Cicadidae	Physiological products	Drying	Diarrhea	Cicada metamorphosis, licorice, jujube	Decocted in water	ZY-2023-Z-014
Lai Ge Bao	Toad	*Bufo gargarizans* (Cantor, 1842)	Bufonidae	Whole	Drying	Treatment of hemorrhoids of the large intestine	Toad, Pig Intestine	Calcined by fire and eaten as a dip	ZY-2023-W-001
Ci Tuan	Hedgehog skin	*Erinaceus aegyptius* (Geoffroy Saint-Hilaire, 1803)	Erinaceidae	Tissue	Drying	Leukorrhea	Hedgehog spines, bamboo roots	Burnt ash and powdered for internal use	ZY-2023-Z-003
Da Xiang Pi	Elephant skin	Elephantidae (Gray, 1821)	Elephantidae	Tissue organs	Drying	Skin ulcers	Elephant skin, snake shed, gypsum	Powdered and applied externally	——
Xiong Dan	Bear Gallbladder	*Ursus thibetanus* (G. Baron Cuvier, 1823)	Ursidae	Tissue organ	Drying	Treating stupor	Used alone	Take with wine	——
Chou Bao	Bovis Calculus	*Bos taurus* (Linnaeus, 1758)	Bovidae	Pathological products	Sulfur fumigation drying or shade drying	Treatment of fever and feverishness in children	Bovine rhubarb, Sichuan rhubarb, cicada shells	Ground and taken internally	——
Niu Dan Zhi	Ox Gall	*Bos taurus* (Linnaeus, 1758)	Bovidae	Tissue organs	Fresh	Trauma causing incontinence	Cow’s bitter gall, Cao Wu	Internal	ZY-2023-Z-004
Shan Yu	Eel	*Muraena alba* (Zuiew, 1793)	Synbranchidae	Entire body	Fresh	Cure distorted eyes and mouth	Used alone	Acupuncture and moxibustion	ZY-2023-W-004
Ji Nei Jin	Galli Gigerii Endothelium Corneum	*Gallus gallus*	Phasianidae	Tissue organs	Dry	Indigestion/Hematochezia/Hypoplasia	Ji Nei Jin	Ground powder for internal use	ZY-2023-W-002
Ji Dan Qing	Eggs	*Gallus gallus*	Phasianidae	Tissue organs	Fresh	Burns	Used alone	External use	ZY-2023-W-006
Pi Ban Chong	Nine Spice Worms	*Coridius chinensis* (Dallas, 1851)	Dinidoridae	Whole	Dry	Hemangioma	Cordyceps sinensis	Rub with juice	ZY-2023-W-005
Lao Hu Gu Tou	Tiger Bone	*Panthera tigris* (Linnaeus, 1758)	Felidae	Tissue organs	Remove sinews, dry	White Bone Pain	Myrrh, tiger’s tibia	Pounded and taken with wine/infused with wine	——
Xiao Tu Gou	Gryllolaptaptidae	*Gryllotalpa spps*	*Grylloidea*	Whole	Dry	Urinary Stones	Gryllotalpa (head removed for bladder stones, head left for urinary stones)	Powdered and swallowed with warm wine	ZY-2023-Z-015
Jia Qiao	Sparrow	Passeres (Linnæus, 1758)	Ploceidae	Whole	Hairless and gutted	Bladder stones	Sparrow	Roasted	ZY-2023-Z-005
Lu Rong	Deer antler	*Cervus canadensis* (Erxleben, 1777)	*Cervus*	Tissue organs	Sliced and dried	Whooping Cough in Elderly	Used alone	Infused in wine or directly consumed	ZY-2023-Z-006
Mi Tang	Honeybee	*Apis mellifera* (Linnæus, 1758)	Apidae	Physiological products	Filtration and removal of impurities	Emptiness Symptoms	Honey/honey, child’s urine (take the middle part)	Apply/take internally	ZY-2023-Z-001
Jiang Chong	Cockroach	*Catharsius molossus* (Linnaeus, 1758)	Blattidae Handlirsch	All	Fresh/dry	Sore Mouth and Tongue/Vomiting Blood, Hemoptysis	Cockroaches, salt	Pound and apply externally	ZY-2023-Z-016
Ha Ma Er	Tadpole	*Rana nigromaculata* (Hallowell, 1861)	Ranidae	All	Drying	Swollen Toxin	Tadpoles, ice chips	Apply with water	ZY-2023-W-007
Chong Shan	Dinosaurus	Lumbricina	Lumbricidae	All	Drying	Mumps	Fresh earthworms	Add sugar to water and take internally	ZY-2023-D-007
Ren Tui	Human fingernails	*Homo Sapiens* (Linnæus, 1758)	Hominidae	Physiological products	Fresh	Pediatric Emergency Fright	Human fingernails, sour jujube seeds	Pound and apply	ZY-2023-Z-017
Shan Yang Jiao	Goat horns	*Naemorhedus goral* (Hardwicke, 1825)	Bovidae	Tissue	Drying	Needle Sticking into Flesh	Wild goat horns, buffalo horns	Ground in water and taken internally	
Yang Nao Zi	Goat brain	*Caprinae*	Bovidae	Tissue organs	Fresh or frozen	Fever and convulsions	Sheep’s brain, Park’s nitrate	Mix the paste well	ZY-2023-D-009
She Dan	Snake Gallbladder	*Dendroaspis polylepis* (Günther, 1864)	Snake Gallbladder	Tissue organs	Drying	Treatment of Pediatric Tumor	Used alone	Take directly	ZY-2023-D-008
Cai She	Cauliflower Snake	*Elaphe carinata* (Günther, 1864)	Colubridae	Whole	Drying	Cough, phlegm and asthma	Used alone	Decocted in water/stewed	ZY-2023-D-006
Long Yi	Snake Molt	*Dendroaspis polylepis* (Günther, 1864)	Colubridae	Physiological products	Drying/Drying	Pediatric eclampsia, dispelling wind	Gypsum, Dragon Bone, Elephant Skin	Ground finely and applied externally	ZY-2023-W-009
Wu She	Ophiopogon	*Ptyas dhumnades* (Cantor, 1842)	Colubridae	Whole	Drying	Skin ulcers	Ophiopogon, Aconite	Smoke therapy	ZY-2023-F-001
Wu Bu She	Herb snake	*Agkistrodon* Palisot de (Beauvois, 1799)	Viperidae	All	Drying	Trembling hands and feet	Mutong, Cow’s Knee	Taken internally or externally in wine	ZY-2023-W-008
Tu Jin	Vipers	*Agkistrodon halys boehmei* Nilson (1983)	Viperidae	All	Drying or fresh use	Rheumatic paralysis	Used alone	Taken internally or in wine	ZY-2023-D-005
Di Shi Po	Rattlesnake	*Armadillidium vulgare* (Latreille, 1804)	Armadillidiidae Brandt	All	Sun-dried or roasted	Treating rheumatic joint pain	Damp Worm	Powdered and applied externally	ZY-2023-D-004
Sha Niu Jiao	Buffalo horn	*Bubalus bubalis* (Linnaeus, 1758)	Bovidae	Tissues and organs	Removing impurities and mashing	Treating pain of tooth decay with holes	Buffalo horn	Swallowing with white wine	ZY-2023-F-002
Ma Huang	Leech	*Hirudo* (Linnaeus, 1758)	Hirudinidae	Whole	Low temperature drying/fresh use	Blood Gonorrhea	Leech, White Cottonwood, Myrrh	Ground into a powder and mixed with warm wine for internal use	ZY-2023-F-004
Luo Si	Snail	Viviparidae (Gray, 1847)	Viviparidae	All	Fresh	Bone fracture pain/bruising (fresh use)	Fresh snail, radish seeds/fresh snail, ginger	Apply externally by pounding/pounding	ZY-2023-Z-011
Di Bie	Earthworm	*Eupolyphaga sinensis* (Walker, 1868)	Carabidae	All	Drying	Umbilical Convexity/Handful of Mouth Wind (Frightening)	Earthworm, Rhizoma Ligusticum, Radix et Rhizoma Chuanxiong, Radix Paeoniae Alba	Add a little wine and decoct	ZY-2023-D-016
Jia Yu Ke	Turtle Shell	*Pelodiscus sinensis* (Wiegmann, 1834)	Trionychidae	Tissue	Drying	Lumbar injury (caused by sprain)	Mountain snail	/Powdered and dried and applied with tung oil	ZY-2023-D-003
Ge Li	Mealybug	*Meretrix meretrix* (Linnaeus, 1758)	Mactra	Tissue	Wash and dry	Uterine Prolapse	Used alone	Infused in wine or powdered for internal use	ZY-2023-Z-009
Wu Gui Ke	Tortoise shell	*Mauremys reevesii* (Gray, 1831)	Emydidae	Tissue	Sun-dried	Nourishing	Tortoise shell, Radix et Rhizoma Dioscoreae	Decoction	ZY-2023-D-010
Wu Zei Gu	cuttlebone	*Sepia officinalis* (Linnaeus, 1758)	Sepiidae	Tissue organs	Drying	Curing pediatric cranial dissolution	Used alone	Powder for external use	ZY-2023-Z-0010
Bai Zu Chong	Centipede	*Scolopendra subspinipes* (Leach, 1815)	Scolopendridae	All	Sun-dried or tumble-dried	Bleeding from trauma	Centipede	Powdered pig’s bile	ZY-2023-W-011
Wu Bei Zi	Cinquefoil	*Schlechtendalia chinensis* (Bell, 1851)	Anacardiaceae	Physiological products	Steamed and dried	Stroke with distorted eyes and mouth	Used alone	Decoction of water/gargle	ZY-2023-Z-012
Ya Dan	Duck Eggs	*Anatinae* (Leach,1820)	Anatidae	Eggs	Fresh	Toothache	Green Shell Duck Eggs, Water Chest	Duck egg decoction	ZY-2023-D-001
Yan Zi Ni	Bird’s Nest Mud	*Hirundo rustica* (Linnaeus, 1758)	Hirundinidae	Mud nests	Remove impurities and mash	Nosebleed	Bird’s Nest Mud	Apply with acid soup	ZY-2023-D-002
Ban Mao	Spanish fly	*Mylabris phalerata* (Pallas, 1781)	Meloidae	Whole	Sun-dried	Testicular Pain	Single use	External use	ZY-2023-W-010
Zhu Ke	Wild Boar Gall Bladder	*Sus scrofa* (Linnaeus, 1758)	Suidae	Tissue organs	Fresh or shade-dried	Skin Diseases	Wild boar’s bile, cypress	Apply	ZY-2023-Z-008
Sha Hou	Golden Sands Cow	Myrmeleontidae (Latreille, 1802)	Myrmeleontidae	Whole body	Powdered	Fire Scald	Bullock, Grateful Parsnip	Internal and external use/pounding mud to wrap the heart of the feet	ZY-2023-D-012
Zhu Niao Bao	Pig Bladder	*Sus scrofa* (Linnaeus, 1758)	Suidae	Tissue	Drying	Stopping drenching and malaria/	Pig’s Bladder	Steam	ZY-2023-D-013
Zhu Yao Zi	Pig Kidney	*Sus scrofa* (Linnaeus, 1758)	Suidae	Tissue	Fresh or refrigerated	Postpartum diaphoresis	Pig Kidney, Pepper	Boiled in water	ZY-2023-W-012
Zhu Ya	Pig’s Tooth	*Sus scrofa* (Linnaeus, 1758)	Suidae	Tissue	Drying	Nocturia	Pig’s Tooth	Burnt ash and drink	ZY-2023-D-015
Zhuo Mu Niao	Woodpecker	Picoides canicapillus	Picidae	All	Dehairing, gutting and drying	Chronic Cough	Woodpecker’s Tongue, Fenugreek	Decoction of water	——
Ye Feng Wo	Beehive	Apis mellifera (Linnæus, 1758)	Apidae	Physiological products	Remove impurities and mash	pediatric eclampsia and snakebite	Single use	Decoction or bath	ZY-2023-Z-007
Jia Pian	Pangolin	Pholidota (Weber, 1904)	Manidae	Tissue organs	Dry in the sun	Cure pediatric cramps	Snake	Apply externally in wine	——
Wa Wa Yu	Ni	*Andrias davidianus* (Blanchard, 1871)	Cryptobranchidae	Tissue organs	Fresh	Skin diseases	Used alone	External use/consumption	——
Tang Lang Dan	Cuttlebone	*Paratenodera sinensis* (Saussure, 1871)	Mantoididae	Tissue organs	Steam scalded and dried	Joint Pain	Cuttlebone, pig’s loin, a cone of incense	Decoction	ZY-2023-D-014

**Fig. 4:**
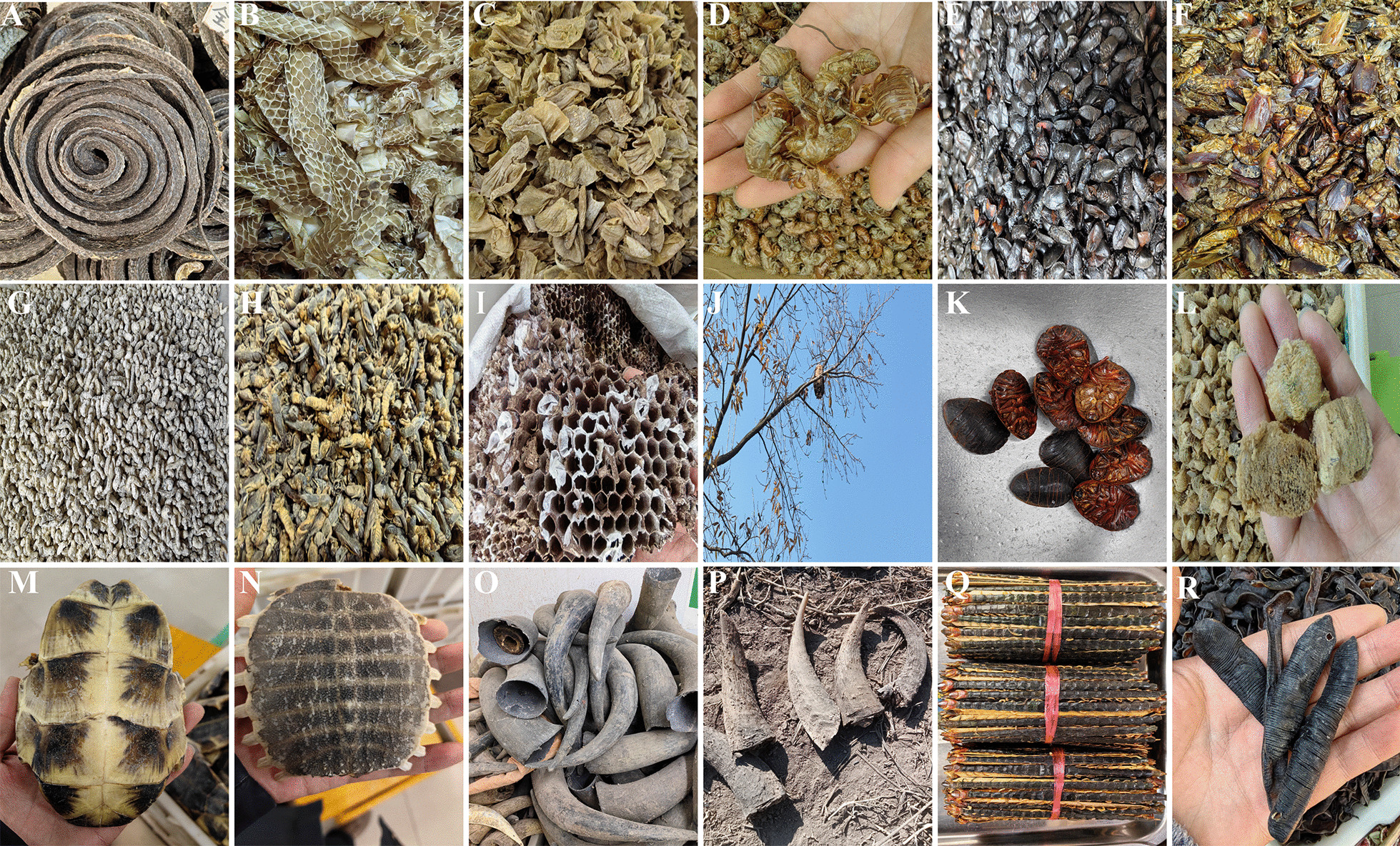
Representative Medicine Specimens. **A** *Ptyas dhumnades* (Cantor, 1842): Commonly known as black-striped snake. **B** Snake Slough: Shed skin of snakes. **C** Inner membrane of chicken gizzard. **D** Cicada Slough: Shed exoskeleton of cicadas. **E** *Coridius chinensis* (Dallas, 1851): A species of beetle used in traditional medicine. **F** *Catharsius molossus* (Linnaeus, 1758). **G** *Bombyx morio* (Linnaeus, 1767): The entire body of the larval stage of the domestic silkworm moth, *Bombyx mori* L., infected by the *Beauveria bassiana* (Bals.) Vuill. **H** Mole Cricket (*Gryllotalpa spps*): A type of cricket used in traditional remedies. **I**, **J** Hives of wild bees. **K** Ground Beetle (*Eupolyphaga sinensis* (Walker, 1868)): Dried bodies of female *Eupolyphaga sinensis* Walker or *Steleophaga* Plancyi (Boleny). **L** Ootheca mantidis (*Paratenodera sinensis* (Saussure, 1871)): Dried oothecae of *Tenodera sinensis* Saussure, *Statilia maculata* (Thunberg), or *Hierodula patellifera* (Serville). **M** Tortoise Shell (*Mauremys reevesii* (Gray, 1831)). **N** Trionyx sinensis Shell (*Pelodiscus sinensis* (Wiegmann, 1834)). **O** Buffalo Horn (*Bubalus bubalis* (Linnaeus, 1758)). **P** Goral Horn (*Naemorhedus goral* (Hardwicke, 1825)). **Q** Centipede (*Scolopendra subspinipes* (Leach, 1815)). **R** Leech (*Hirudo* (Linnaeus, 1758))

The majority of these medicinal products are derived from locally sourced animals. However, certain items, like Elephant skin, are not presently traceable to local origins. These animal-derived medicines significantly influence the daily lives of the Gelao community. Notably, many of these materials are integral to the Gelao’s traditional diet, including some now-banned animals like Bears and Tigers, previously relished as delicacies. This is similarly true for certain snakes; despite stringent protection measures, the Gelao still procure various snakes for consumption, medicinal purposes, or liquor infusion. Presently, the Gelao mainly utilize animal-derived medicines comprising non-strictly protected insects and derivatives of domestic animals, occupying a vital position in their traditional medicinal practices and cultural heritage (Fig. 4).

### The utilization and production of animal-derived medicines among the Gelao community

The Gelao community extensively utilizes a diverse range of animal-derived medicines, including insects, mollusks, reptiles, fish, amphibians, birds, mammals, and an assortment of physiological and pathological products sourced from these creatures. The procurement of these medicinal animals is multifaceted: Wild Capture (Leveraging the natural environment, the Gelao community utilize a variety of methods to sustainably capture wild animals for both medicinal and culinary applications). Domestic Breeding (In addition to food production, the Gelao engage in the breeding of animals, harnessing their blood, meat, skin, bones, horns, and certain organs and secretions for medicinal purposes). Market Purchase (Animal medicines are frequently acquired at rural markets, typically sold by seasoned herbalists, and are widely used within the farming community for the treatment of prevalent ailments).

The Gelao community adheres to specific standards and methodologies in the preparation and application of animal-derived medicines. Their processing techniques include a series of steps such as killing, cleaning, drying, crushing, decocting, soaking, and grinding. These medicines are processed variably, contingent on their inherent properties and intended uses. For example, insects are generally scalded and dried to expedite dehydration and avert egg hatching, akin to the treatment of gallnuts; physiological and pathological products, like silkworm feces and cicada slough, are typically cleaned and directly dried. Valuable substances, such as bear and snake bile, are often air-dried to preserve their medicinal virtues, in contrast to larger animals which are eviscerated before drying.

The Gelao community demonstrates extensive knowledge and established principles in the dosing and combination of animal-derived medicines. Dosages are meticulously tailored based on the severity of the condition, the patient’s age, and their overall health, typically prescribed one to three times daily, with each dose ranging from three to ten grams. The composition of these medicines follows the traditional ‘Monarch, Minister, Assistant, and Guide’ principle, which considers their properties, effectiveness, and channel tropisms, as well as their potential synergistic or antagonistic effects, to enhance therapeutic efficacy and minimize side effects. The selection and application of these medicines vary markedly among practitioners, reflecting their individual experience and expertise. The administration methods are customized according to the specific nature and intended use of the medicines, encompassing oral routes (including decoctions, tinctures, powders, pills, and pastes), topical applications (such as direct application, patches, washes, and fumigation), and inhalation methods, particularly for respiratory ailments, headaches, and toothaches.

**Fig. 5:**
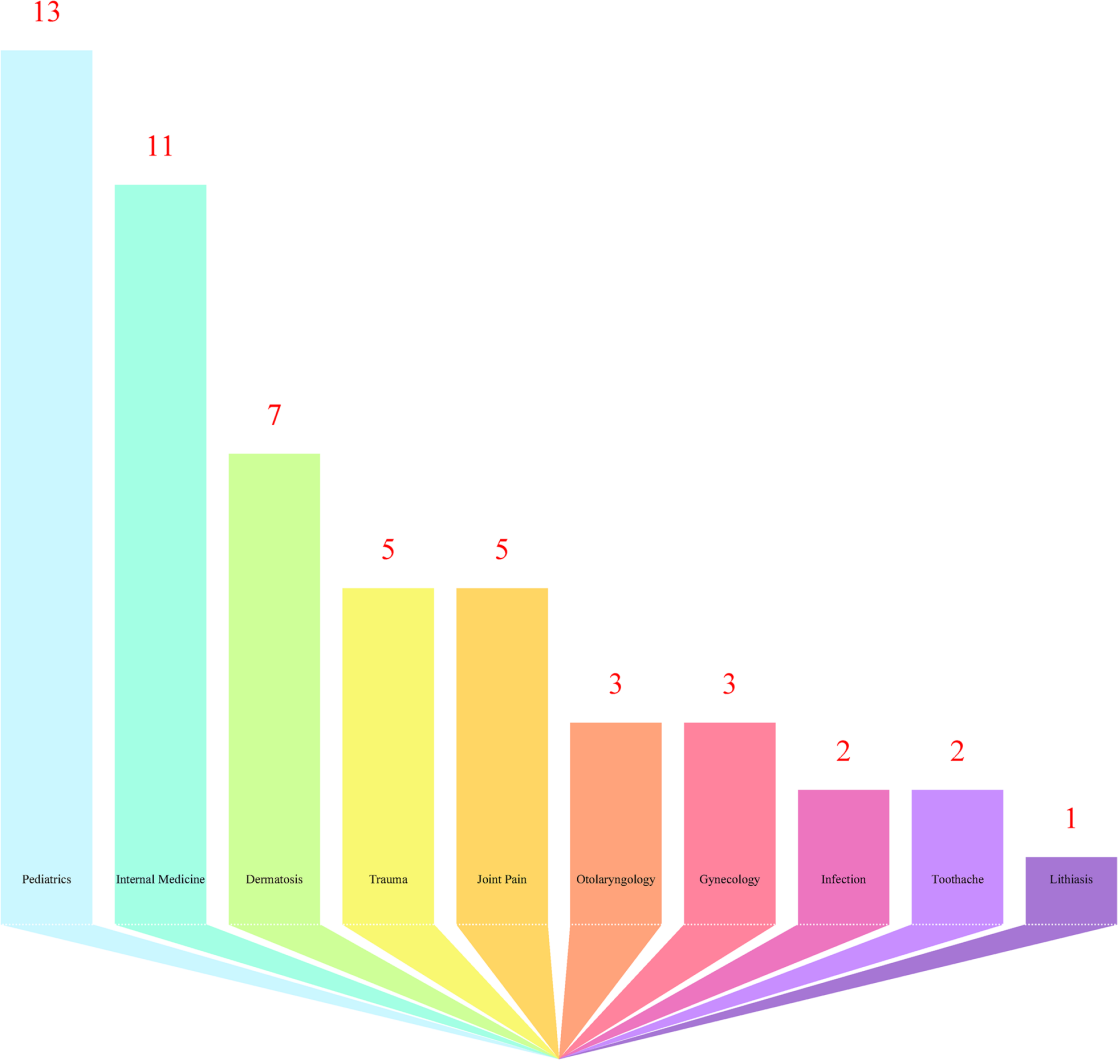
Efficacy of animal-derived medicines used by the Gelao community

Our survey catalogued 55 traditional medicinal remedies exhibiting a broad spectrum of effects and indications (Table 3), primarily distributed across ten categories: 13 for pediatric disorders, including silkworm pupae for food stagnation and cicada slough for diarrhea in children; 11 for internal medicine conditions, such as stiff silkworm for cough and asthma; 3 for gynecological issues, like soft-shelled turtle shell for uterine prolapse; 7 for dermatological conditions, including elephant skin for skin ulcers and blister beetles for various skin diseases; 3 for otorhinolaryngological disorders, such as eel for facial paralysis; 5 for trauma care, like cuttlefish bone for bleeding injuries; 5 for alleviating pain in the neck, shoulders, waist, and hip joints, such as viper for rheumatic joint pain; 1 for urinary and bladder stone disorders, like mole cricket; 2 for infectious diseases, such as cockroach for swelling and toxicity; and 2 for dental pain, like gallnuts for toothaches. Additionally, some medicines are primarily used for nourishment and the treatment of other specific diseases (Fig. 5).

**Table 3:** Comparison of traditional usage of mineral medicines by the Gelao people with other ethnic applications and modern pharmacological research

Herbs	Representative Chemical Component	Bioactivity	Representative drugs	Use of ethnicity
Can Yong	Protein, Amino acids, Protein fiber	Anti-tumor, Anticancer, Hypoglycemic, Antibacterial	Silkworm Chrysalis Kidney Tonic Capsule, Kidney and Liver Ning Capsule	Han, Tu Jia, De Ang, Jing Po
Bai Jiang Can	Flavonoids, Polysaccharides, Beauveria	Anticoagulant, Anticonvulsant, Hypolipidemic, Hypoglycemic	Quick-acting Cough Suppressing Soup, White Stiffy Drink, Eclampsia Capsule, Elevating and Lifting Scattering	Han, Bai, Chao Xian, Miao
Can Sha	Phenols, Flavonoids, Terpenes and Polysaccharides	Hypoglycemic, Improves pancreatic islet function	Silkworm Sand Bullet, Blood Shengning Tablet	Han, Wei Wu Er
Chan Tui	Chitin, Amino acids and Trace elements	Anti-inflammatory, Sedative analgesic antispasmodic, Anticonvulsant	Anti-fungal and anti-itching granules, Dampness toxin clearing capsule, Wu Hu chasing the wind powder	Han, Yi
Chan Chu	Toadstoolene, Indole alkaloids	Anti-tumor, Anti-hepatitis B virus, Immunomodulation	Huachansu Oral Liquid, Toad Crisp Injection	Han, Yi, Wa, Miao, Bu Yi, Tu Jia, Ha Ni, Na Xi
Ci Wei	Hedgehog Proteins	Promote osteoclast differentiation	Hedgehog Skin Pill, Prostate Bixi	Han, Wei Wu Er, Yi
Da Xiang Pi	Creatine, Agonist proteins, Vitamins	Anti-inflammatory, Analgesic, Accelerates wound healing	Elephant skin and astragalus soup, elephant skin and muscle cream	Han, Dai
Xiong Dan	Bile acids, Amino acids, Proteins	Anti-inflammatory, Antimicrobial, Antiviral, Anti-tumor, Hepatoprotective, Anti-thrombotic, Anti-fatigue	Compound Bear’s Gallbladder Granules, Bear’s Gallbladder Pill Capsules, Bear’s Gallbladder Heart-Saving Pill	Han, Meng Gu, E Lun Chun, Yi, Zang
Niu Huang	Ursodeoxycholic acid, Taurine goose deoxycholic acid	Inhibition of inflammatory mediator release, Immunomodulation	Niu Huang Antihypertensive Pill, Angong Niu Huang Pill, Niu Huang Antidote Tablet	Han, Wei Wu Er, Zang, Meng Gu, Qiang, Yi, Tu Jia
Niu Dan Zhi	Bile acids, Cholesterol, Fatty acids	Suppresses chronic inflammation, Strengthens immunity	Gallbladder Clearing Capsule, Cataract San, Eight Treasures Five Gallbladder Medicine Ink	Miao, Wa, Zang
Huang Shan	DHA, Lecithin, Eelsin, Protein	Improves memory, Lower blood sugar, Improves eyesight	Gold Eel Thirst Elimination Granules	Tu Jia, Wa, Dong, Yao, Yi, Ha Ni, Chao Xian, Bu Lang, Ji Nuo
Ji Nei Jin	Protein, Amino acid, Trace element	Promote gastric secretion, Digestion, Stone removal	Longmu Bone Strengthening Granules, Compound Chicken Neijin Tablets, Stomach Kang Capsules	Dai, Wei Wu Er
Ji Dan	Protein, Egg white peptide, Whey protein	Antioxidant activity, Promote mineral absorption, Lowers blood pressure	Ginseng and Antler Oral Liquid, Stomach Kang Capsules	Dai, Yi, Tu Jia
Jiu Xiang Chong	Crude protein, Fat, Vitamin, Amino acid	Anti-tumor, Antibacterial, Anti-inflammatory	Kang Lixin Capsules, Cancer Pain Capsules, Liver-Sparing and Yang-Expanding Capsules	Han, Miao, Dai, Bu Yi, Tu Jia
Hu Gu	Collagen, Osteomucin, Polysaccharides	Anti-inflammatory, Sedative, Analgesic	Musk Tiger Bone Cream, Tiger Bone Papaya Pill, Bone Strengthening Pill	Han, Yi, Dai, Na Xi
Lou Gu	Amino acids, Alkaloids, Anthraquinones, Fatty acids	Diuretic, Sedative, Antibacterial	Gryllotalpa crickets, Prostate Bixi Capsules	Han, Miao, Bu Yi, Shui, Yi
Ma Que	Low fat, High protein	Strengthening the kidney and Yang, Benefiting the essence, Warming the waist and knees	Sparrow Soup, Cordyceps Sparrow Soup, Bird’s Nest Porridge	Han, Zhuang, Zang
Lu Rong	Antler peptides, Sugars, Amino acids	Anti-fatigue, Immunomodulation, Anti-aging Antioxidant, Hypoglycemic	Deer antler tonic soup, deer antler capsule, ginseng and deer antler pill	Han, Chao Xian, E Lun Chun, Dai, Meng Gu
Mi Feng	Honeybee Spleen, Royal Jelly, Bee Pupa	Antioxidant, Antibacterial, Anti-inflammatory and analgesic	Rhinitis Granules	Han, Wa, Shui, Zang, Yi, Miao, Bu Yi
Zhang Lang	Polypeptides, Alcohols, Polysaccharides, Chitin	Antioxidant effect, Antimicrobial effect, Tumor cell inhibition, Diuresis	Mantis Pill, Rehabilitation New Liquid	Yi, Dai
Ke Dou	Inorganic elements, Amino acids	Clearing heat and removing toxins, Treating feverish sores and swellings, Anti-tumor	Tadpole Poison Extractor	Han, Yi
Di Long	Fatty acids, Lipids, Proteins, Nucleotides	Clearing heat and removing toxins, treating feverish sores and swelling, anti-tumor	Di Long San, Compound Di Long Capsule, Chicken Embryo Di Long Cream	Zhuang, Wei Wu Er, Dai
Ren Zhi Jia	Keratin, Fatty acids, Amino acids	Anti-tumor, Antioxidant, Cough suppressant and asthma relief	Laryngitis Pill, Xizi San	Tu Jia
Shan Yang Jiao	Keratin, peptides, Amino acids, Inorganic salts	Sedative, Analgesic, Anticonvulsant, Central inhibitor	Niuhuang Qingbian Kaijiao Pill, Compound Yangjiao Tablet, Dendrobium Nightshade Pill	Han, Zang, Wei Wu Er, Dai
Yang Nao	Peptides, Ascorbic acid, Niacin, Riboflavin	Antioxidant, Treats wind-cold in the brain, Headache	Goat’s Brain Ointment, Goat’s Brain Decoction, Yuhua Dan	Meng Gu
She Dan	Bile acids, Bile pigments, Cholesterol	Anti-inflammatory, Immunomodulatory, In vitro antimicrobial, Antihypertensive effects	Snake Gallbladder and Chuanbei Liquid, Snake Lian and Chuanbei San, Ox-Huang Snake Gallbladder and Chuanbei Liquid	Ha Ni, Tu Jia, Yi, Dai
Wang Jin She	Serum of *Elaphe carinata* (Günther)., Snake molt extract from *Elaphe carinata* (Günther)	Anti-snake venom, Antibacterial	Three Snakes Rheumatism Liquor, Tincture of Analgesia and Vitalizer	E Lun Chun, Ha Ni, Wei Wu Er
She Tui	Collagen, Amino acids, Fatty acids, Sterols	Antibacterial, Anti-inflammatory	Dermatological Blood Poison Pills, Dermatological Blood Poison Tablets	Han, Yi, Wei Wu Er
Wu Shao She	Amino acid, Protein, Nucleoside components	Treating rheumatic paralysis, Tetanus and leprosy	Wantong Tendon and Bone Tablet, Three Snakes Rheumatism Wine	Han, Meng Gu
Qi She	Protein, Amino acids, Phospholipids, Nucleosides	Dispel wind, Promote circulation, Antispasmodic	Compound Herb Granules, Compound Herb Capsules, Golden Dragon Capsules	Han, Dong, Shui, Yao, Tu Jia
Fu She	Cholesterol, Taurine, Fat, Lipid	Anti-inflammatory, Anti-thrombotic, Anticancer	Astragalus Vipers Capsules, Vipers Jade Bamboo Capsules	Chao Xian, Shui, Yao, Zang, E Lun Chun
Shu Fu	Taurine, Glucosamine, Aminoglycans	Analgesic, Anti-inflammatory, Inhibiting inflammatory pain	Ginseng and turtle shell decoction pill, turtle shell decoction pill, red gold elimination capsule, elimination of lactation capsule	Han, Wa
Shui Niu Jiao	Mercaptopeptides, Keratin, Amino Acids	Antipyretic, Sedative, Anti-Inflammatory, Hemostatic	Buffalo Horn Antidote Pills, Qingkailing Capsules, Twenty-five-flavored Mabao Pills	Dai, Wa, Yao, Bu Lang, A Chang, Dong, Ha Ni, Ji Nuo, Jing Po, Mao Nan, Meng Gu, Zang
Shui Zhi	Proteolytic peptides, Hirudin, Kissing hirudin	Anti-coagulation, Anti-thrombosis, Anti-tumor, Anti-atherosclerosis	Brain Heart Capsules, Tongxinluo Capsules, Astragalus Capsules	Han, Yi, Wa, Zhuang, Tu Jia
Luo Si	Amino acids, Minerals, Trace elements	Gangrene, Edema, Dysuria, Hemorrhoids and blood in stools	Compound Anti-Hemorrhoid Suppository	Han, Ha Ni, Wa, Miao, Bu Yi, Shui, Yi
Tu Bie Chong	Amino acids, Volatile oils, Alkaloids, Active peptides	Dissolving thrombus, Anticoagulant, Hypolipidemic, Antioxidant, Tumor inhibitor	Elderberry Seven Cents Tablet, Soft Vein and Plaque Granule, Stroke Rejuvenation Tablet	Zhuang, Miao, Yao, Zang, Chao Xian, De Ang, Jing Po, Tu Jia
Bie Jia	Vitamin D, Turtle shell polysaccharide, Keratin	Anti-hepatic fibrosis, Anti-lung fibrosis, Anti-tumor, Immune regulation	Artemisia Artemisiae Tetraptera Soup, Compound Tetraptera Soft Liver Tablets, Tetraptera Decoction Pills	Han, Zhuang, Miao, Yi, Shui Yao, Dai, Tu Jia, Meng Gu, Chao Xian
Ge Jie	Carotenoids, Lipids, Polysaccharides, Proteins	Anti-tumor, Anti-coagulation, Antibacterial, Anti-virus	Mealybug Pills, Mealybug Capsules, Ginseng and Astragalus Mealybug Paste	Han, Yao, Zhuang, Dai, Yi, Wei Wu Er
Gui Jia	Amino acids, Collagen, Trace elements	Enhance immunity, Promote development, Delay aging	Turtle shell powder, Turtle gum yin nourishing granules, Turtle shell yin nourishing tablets	Han, Zhuang, Dai
Hai Piao Shao	Calcium carbonate, Peptides, Chitin, Chitosan	Hemostasis and coagulation, Antibacterial, Antioxidant, Immunomodulation	Yuanhu Stomach Shuo Capsule, Stomach Shuning Granule, Fast Stomach Tablet	Han, Zhuang
Wu Gong	Proteins, Peptides, Fatty acids, Quinolines	Anti-tumor, Anti-thrombosis, Anti-cardiac ischemia, Antibacterial, Analgesic and Anti-inflammatory	Anti-Embolism Capsules, Stroke Rejuvenation Tablets, Xuanqi Tongpao Capsules	Han, Tu Jia, Wa, Zhuang, Yi
Wu Bei Zi	Tannins, phenolic acids, amino acids, trace elements	Anti-caries, Antibacterial, Hemostatic, Anti-inflammatory, Anticancer, Antioxidant	Wu Huang Cream, Wu Miao Shui Xian Cream, Shu Ling Throat Tablets	Miao, Bu Yi, Shui
Ya Dan	Protein, Amino Acids, Fatty Acids, Vitamins	Enhancement of immunity	Duck Egg Soup	Bu Yi, Dai
Yan Wo Ni	Protein, Sugar, Fat, Vitamin	Antioxidant, Anti-aging, Anti-virus, Immunomodulation	Green Bird’s Nest Dan, Compound Bird’s Nest Tablets	Wa, Zang
Ban Mao	Zanthoxylin, fat, bound zanthoxylin	Antiviral, Anti-tumor, Immune regulation	Compound Spotted Fish Capsules, Activator Capsules, Panax Ginseng Cream, Shu Tendon Pain Relieving Capsules	Han, Zang, Meng Gu, Wei, Yi, Wa
Ye Zhu Dan	Bile acids	Smoothing Qi and regulating the lungs, Resolving phlegm and eliminating asthma, Postpartum weakness	Roasted Wild Boar Gallbladder in Warm Wine for Postpartum Pink Scald	Yi, Dai
Zhu Pao	UBM, Collagen type I, Laminin, Glycoproteins, Growth factors	Promoting wound repair, Pediatric enuresis, Frequent urination in the elderly, Urinary incontinence	Stewed Pig’s Bladder with Sansi, Stewed Pig’s Bladder with Morinda citrifolia and Walnut, Stewed Pig’s Bladder with Plantain	Han, Bu Yi
Zhu Shen	GTF, Thyroid inhibitors	Kidney deficiency, Postpartum abdominal pain	Pig Kidney Porridge, Pig Kidney Soup, Magnetite Pig Kidney Soup	Han, Chao Xian, Wa
Zhu Chi	–	Mental disorder, Fever	–	Bu Lang, Ha Ni, Ji Nuo
Zhuo Mu Niao	–	–	–	–
Feng Fang	Amino acids, Peptides, Organic acids, Phenols	Anti-inflammatory, Antimicrobial, Anticancer, Antioxidant, Anti-ulcer, Antiviral	Stomatitis spray, anti-embolism capsule, musk rheumatism capsule	Han, Yi, Dai
Chuan Shan Jia	L-silk-L-tyrosine cyclic dipeptide, Cyclic dipeptide, Nucleosides	Analgesic activity, Promoting milk protein synthesis, Hypoxia resistance, Activating blood circulation and removing blood stasis	Sanjia San, Dokkaku Paste, Musk Huiyang Paste	Yi, Na Xi, Dai, Wei Wu Er
Da Ni	Collagen, Arachidonic acid	Lowering blood sugar, Lowering blood lipids, Lowering blood pressure, Enhancing immunity, treating burns	Giant salamander ground into dry powder mixed with tung oil for burns	Miao
Sang Piao Shao	Proteins, Amino acids, Sugars, Lipids	Anti-fatigue, Enhance immunity, Anti-lipid peroxide, Anti-diuresis	Wuji Baifeng Pills, Gendi Granules, Nocturia Pills	Han, Yi, Shui, Dong, Tu Jia
Jin Sha Niu	Long-chain fatty acids, Fatty acid esters, Peptides	Anti-thrombotic and Anticoagulant, Hypoglycemic, Analgesic and Anti-inflammatory, Tissue repair	Jinsha Niu Fossilized Stone Tablet, Si Jin Tang	Han, Wa, Ha Ni

### Quantitative evaluation of animal-derived medicines used by the Gelao community

**Fig. 6:**
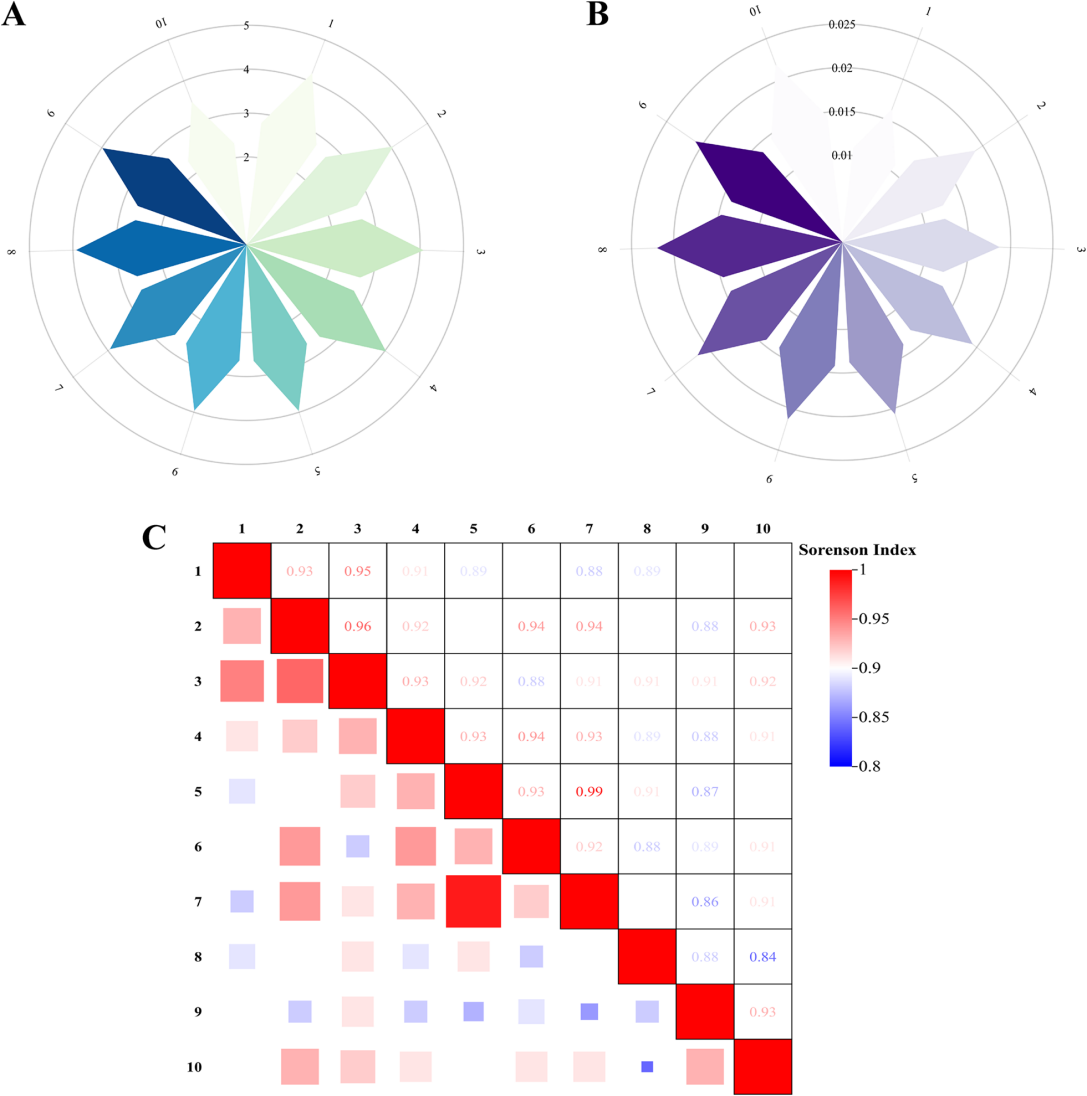
Analysis of animal-derived medicines usage in different Gelao villages. **A** Simpson Index: evaluates the uniformity of the usage of animal-derived medicines among different Gelao villages. **B** Shannon–Wiener Index: assess the richness of information regarding animal-derived medicinals used by the Gelao people across different villages. **C** Sorenson Index: evaluate the similarity of the medicinal usage information across different Gelao villages

In this study, we sought to assess the diversity, significance, and uniformity in the use of animal-based medicines within the Gelao community. Data gathered from 10 villages were analyzed, focusing on the concepts of evenness, richness, and similarity in medicinal knowledge. Evenness, which denotes the distribution of medicinal information, and richness, referring to the total amount of such information, are key indicators in this context. The Shannon Wiener Index (*H*′) was used to measure evenness, and the Simpson Index (*D*) for richness, as illustrated in Fig. 6A and B. The Shannon Wiener Index showed variations from 3.4873 to 4.1883, and the Simpson Index from 0.0163 to 0.0220, demonstrating high levels of both evenness and richness in the Gelao’s use of animal-based medicines, indicative of their extensive knowledge and application of such resources. Notably, Village 1 had the highest Shannon Wiener Index (4.1883) and the lowest Simpson Index (0.0163), implying a well-distributed and least concentrated knowledge base, suggesting a balanced dissemination of information. Conversely, Village 10, with the lowest Shannon Wiener Index (3.4873) and the second highest Simpson Index (0.0218), exhibited the least dispersion and highest concentration of knowledge, indicating an unbalanced distribution. Overall, barring Village 1, the evenness of information among the remaining nine villages was relatively consistent, reflecting a shared understanding in the use of animal-based medicines across these Gelao communities.

To evaluate the similarity of medicinal knowledge across the villages, the Sorenson Index (CS) was employed. This index measures the degree of shared knowledge among communities, with a higher index signifying more similarity in medicinal practices. As shown in Fig. 6C, the Sorenson Index ranged from 0.84 to 0.99, indicating a significant uniformity in the data across the 10 villages. This uniformity likely stems from the closely connected nature of the Gelao communities in northern Guizhou. The highest similarity was observed between Villages 5 and 7 (0.99), which are geographically close, whereas the lowest was between Villages 8 and 10 (0.84), further apart, suggesting that geographical proximity influences the homogeneity of medicinal knowledge among the Gelao.

**Fig. 7:**
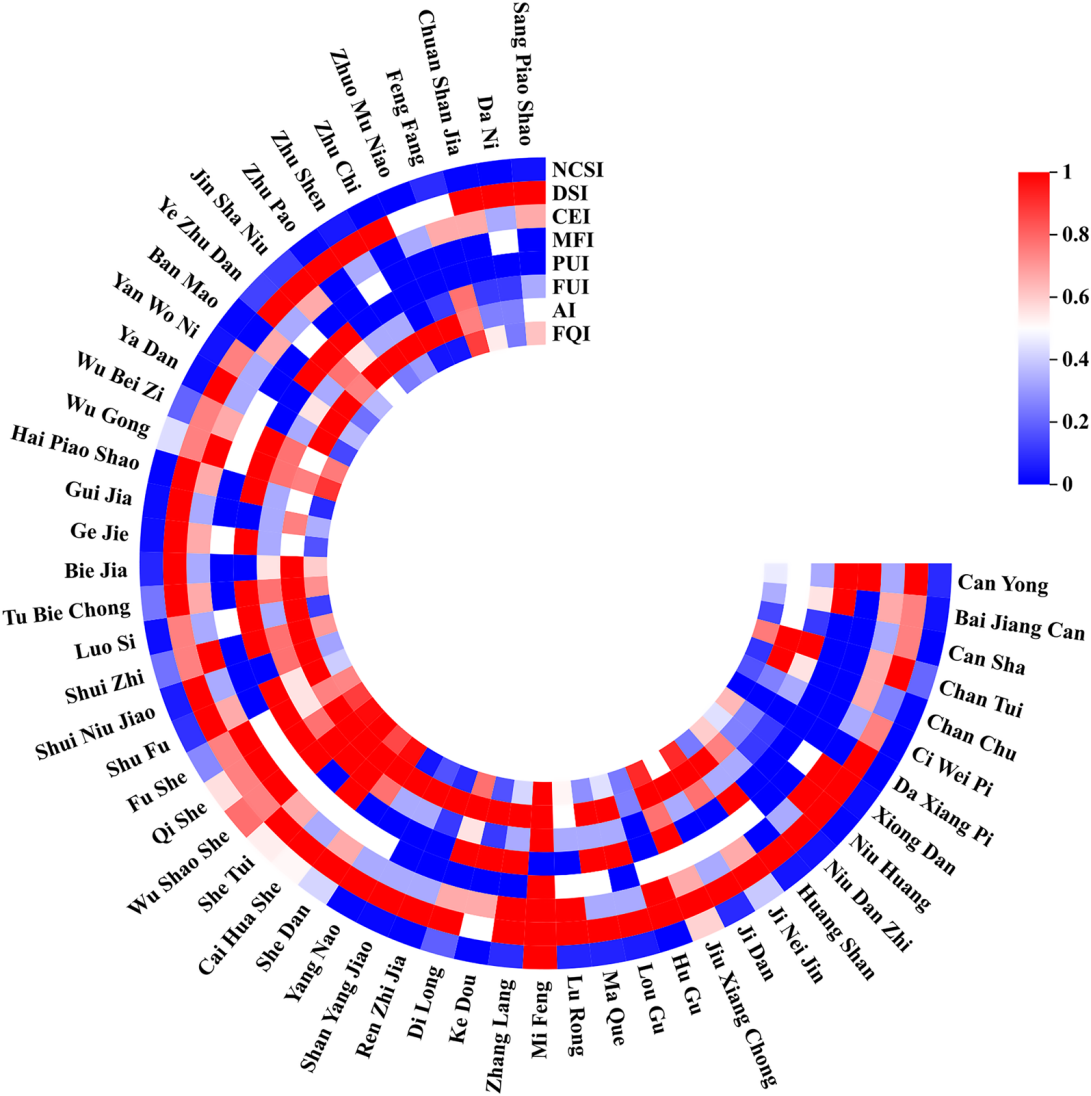
Importance Indices for Animal-Derived Medicinals Used by the Gelao community. (FQI is the frequency of quotation index. AI is the availability index. FUI is the frequency of utilization index. PUI is the parts used index. MFI is the multifunctional use index. CEI is the curative effect index. DSI is the drug safety index. NCSI is the national cultural significance index)

To assess the cultural significance of animal-derived medicines in the Gelao community, we implemented the National Cultural Significance Index (NCSI). This index gauges the role and relevance of these medicines in traditional Gelao healthcare, with a higher NCSI denoting greater significance and acceptance. Our analysis encompassed 55 animal-derived medicines traditionally utilized by the indigenous Gelao residents. Figure 7 presents the NCSI comparisons of these medicines, allowing us to categorize them based on their usage frequency, value, and role in local healthcare practices.

Medicines ranking highest in importance (NCSI > 500) comprise 9 varieties, including honey, cinnabar, Blister Beetles, Chicken gizzard, and various snake medicines. These share traits such as regional availability, proven effectiveness, and relative safety, continuing to be integral to the Gelao community’s health regimen, with many being consumable. The second tier (500 > NCSI ≥ 100) features 16 types like Ground Beetles, gallnuts, cicada slough, etc., frequently used in traditional Gelao medicine, though less often due to their limited culinary applications. The third category (100 > NCSI ≥ 10) includes 19 diverse types, exemplified by medicines like mole cricket, water buffalo horn, etc. The final tier (10 > NCSI) encompasses 11 types, characterized by infrequent use, toxicity, or prohibition, often involving protected species.

**Fig. 8:**
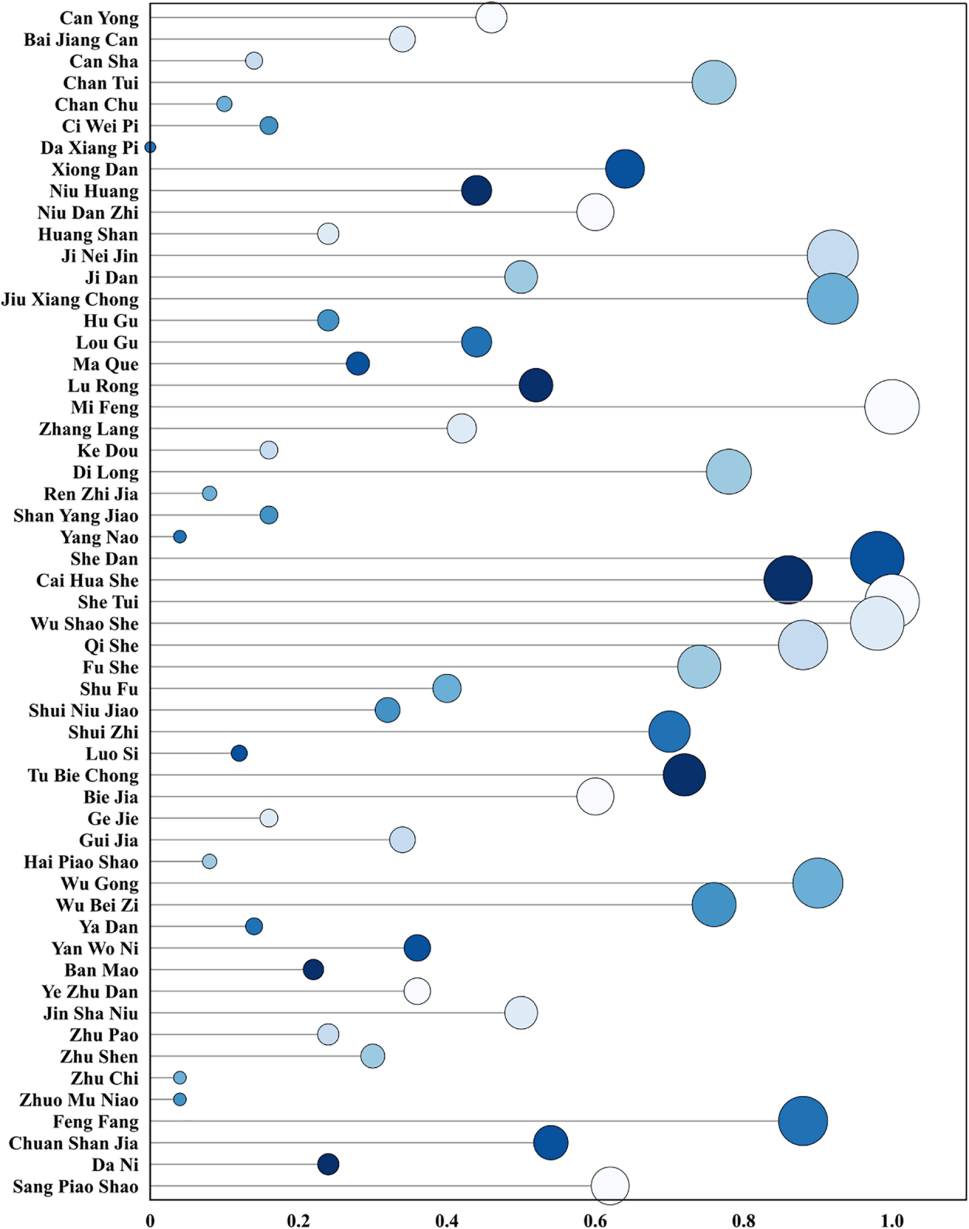
The utilization frequency evaluation of animal-derived medicines among the Gelao people. (The horizontal axis quantifies the Utilization Frequency (HUF) of animal-derived medicines, representing the number of times different medicinal substances have been documented in use within the community. The vertical axis enumerates the variety of animal-derived medicinal substances employed, underscores the reliance on and diversity of animal resources in the traditional medical practices of the Gelao people.)

The Utilization Frequency (HUF) of medicines ranges from a minimum of 0 to a maximum of 1. The medicinal with the lowest HUF, at 0, is Elephant Skin, for which we obtained only minimal information from the literature. None of the 50 informants in our field study provided any related information. We specifically inquired about Elephant Skin with the local residents, and all informants indicated a lack of familiarity with this medicinal. The highest HUF value, at 1, indicates unanimous reporting by all informants, which was the case for Honey and Cicada Slough. The widespread practice of beekeeping among local residents accounts for the high frequency of Honey, while the abundance of Cicada Slough may be related to the local climate being humid and the forests dense, providing a rich resource of snakes (Fig. 8).

### Investigating the use and pharmacological research of animal-derived medicines by the Gelao community

Our research delved into the properties, activities, and applications in Traditional Chinese Medicine and other ethnic practices of animal-derived medicines traditionally employed by the Gelao. Through a literature review of 55 traditional Gelao medicines (Table 3), we discovered unique ethnic attributes in these remedies, demonstrating the Gelao’s profound understanding and use of natural resources. Despite some influences from Traditional Chinese Medicine and modern therapeutics, these medicines maintain their distinctive ethnic identity. Among them, 37 (67.3%) are listed in the ‘Chinese Pharmacopoeia,’ with the remaining 18 (32.7%) being exclusive to the Gelao or shared with other ethnic groups, including medicines like woodpeckers, centipedes, scorpions, eels, Chicken gizzard, and leeches. This highlights the Gelao’s unique approach to using animal-derived medicines and their cultural exchanges with other ethnicities.

A comparative analysis of the Gelao’s traditional medicinal applications and their modern clinical uses revealed a scientific basis for their ethnically unique treatments. Among the 55 medicines, 38 (69.1%) have pharmacological properties and clinical applications aligning with the treatments prescribed by Gelao doctors. For instance, Lin et al. [52] achieved better therapeutic effects in treating facial paralysis using acupuncture and external application of eel blood compared to acupuncture alone, consistent with the Gelao’s use of eel for treating facial asymmetry. The Gelao use Chicken gizzard to treat indigestion, and Yang et al. [53] found that its application in children with gastrointestinal syndrome primarily presenting with diarrhea had a good clinical effect, being fast-acting and requiring a short treatment period, related to its ability to improve gastrointestinal function. The Gelao’s use of Cow bezoar and Snake gallbladder to treat children’s colds, coughs, and fevers is related to their anti-inflammatory and sedative properties [54]. Using leeches for treating pain from bone fractures is related to the anticoagulant activity of hirudin in leeches, which effectively eliminates blood stasis [55].

The pharmacological activities of 17 medicines (30.9%) differ from their traditional Gelao applications. Some of these have been confirmed by modern pharmacological research for their active components and mechanisms of action. For example, hedgehogs are used for peptic ulcers [56] and burns [57], but the Gelao use them for treating leukorrhea, and the anti-inflammatory properties of hedgehog skin could be related to treating these conditions, indicating these medicines have unique potential research value. Additionally, we compared representative patent medicines for the 55 medicines, finding that 44 (80%) of these medicines’ treated symptoms were consistent with Gelao doctors’ applications, while 11 (20%) had slight discrepancies. This also illustrates the scientific nature of the Gelao doctors’ medication practices.

The traditional application of animal-derived medicines by the Gelao is an important part of their cultural heritage, a precious asset accumulated and passed down through long-term production and life practices. These medicines not only reflect the Gelao’s understanding and utilization of nature but also their interaction and borrowing from other ethnic groups. The material basis, biological activity, and application in Traditional Chinese Medicine and among other minorities of these medicines warrant further research and development, to promote the development and protection of Gelao ethnic medicine.

## Discussion

Animal-derived medicines in China have a rich and extensive history, documented over millennia in traditional Chinese medicine texts [14, 15]. The role of animal-derived medicines in Gelao traditional medicine, compared to the traditional medicines of other ethnic groups worldwide, has its unique characteristics and values but also faces challenges and predicaments that need our attention.

### The status of animal-derived medicines in Gelao community

The role of animal-derived medicines in Gelao traditional medicine is closely related to their geographical environment, ecological resources, cultural traditions, and socioeconomic factors. The Gelao populace predominantly resides in the Wuchuan Gelao and Miao Autonomous County, Daozhen Gelao and Miao Autonomous County of Guizhou Province, and Wulong County in Chongqing City. The area is characterized by a subtropical humid monsoon climate, complex terrain and landscape, and rich water and biological resources, providing an excellent environment for the growth of plants and animals, and serving as an important basis for the use of animal-derived medicines by the Gelao Community [58]. The Gelao have a long history and culture, where the worship and utilization of animals form an essential part [59]. The Gelao traditionally believe in animism, considering animals as incarnations or messengers of spirits with magical powers and functions, capable of blessing people’s health and happiness, and treating diseases and disasters [60]. The Gelao’s selection and utilization of medicinal animals reflect their profound understanding and unique insights into animals, as well as their respect and appreciation, representing significant ethnobiological value [61].

Furthermore, the standing of animal-derived medicines within Gelao traditional medicine is informed by a global perspective, influenced by the practices of other ethnic groups’ traditional medicines. Compared to plant-based medicines, animal-derived medicines are at an absolute disadvantage in terms of variety and application [62]. Particularly, the perception of safety in animal-derived medicines by traditional Chinese medicine and modern medicine has put them in an awkward position [63]. Although we have gathered rich traditional knowledge about the use of animal-derived medicines from the Gelao, in practice, we observed a limited variety and quantity of such medicines in Gelao doctors’ clinics or herbal stalls during our investigation. On the one hand, this is related to the fact that the variety of medicines animals can provide is far less than plants—the third national survey of Chinese medicinal resources collected 12,807 medicines, with plant medicines accounting for over 89%, and animal medicines only about 10% [64]. On the other hand, the difficulty in obtaining wild animals due to wildlife protection laws and regulations is a significant reason for the reduced use of animal-derived medicines by the Gelao. Additionally, the development and popularization of modern medicine have impacted and challenged the status of animal-derived medicines in Gelao traditional medicine. Questions surrounding the efficacy and safety of certain animal-derived medicines, alongside the emergence of substitutes and novel pharmaceuticals, have led to a decline in their demand and utilization in Gelao traditional medicine.

The status of animal-derived medicines in Gelao traditional medicine has its unique characteristics and values but also faces challenges and predicaments. We need to understand and evaluate this from multiple angles and levels. We should not oversimplify; neither blindly reject and deny, nor blindly praise and imitate. Instead, based on respecting and protecting the traditional knowledge and culture of the Gelao Community, we should conduct in-depth research and development of animal-derived medicines in Gelao traditional medicine using scientific methods and means to explore their potential values and roles.

### Traditional knowledge and utilization of animal-derived medicines in the Gelao community

The Gelao community’s utilization of animal-derived medicines, both in terms of species richness and the richness of traditional knowledge, is far less extensive compared to plant medicines. However, the information they provide displays distinct ethnic characteristics. The use of animal-derived medicines among the Gelao Community is more mysterious compared to the more scientifically understood plant medicines. These practices include the fresh application of animal parts, incorporation into dietary therapy, and preparation through alcohol infusion, highlighting a unique blend of tradition and empirical wisdom.

Our investigation and analysis indicate that the Gelao’s use of animal-derived medicines involves a wide variety of animal, reflecting their extensive utilization and deep understanding of animal resources. The methods of utilizing these medicines are diverse, including not only common methods like direct consumption, decoction, and grinding but also special methods such as ashing and burning the materials, and then consuming with water or in combination with other medicines. This process transforms organic substances into inorganic ones, enhancing the efficacy and stability of the medicines, reducing animal odors and impurities, and increasing convenience and comfort in consumption. Additionally, different degrees of burning produce medicines with different properties, such as those burned to white ash generally having cooling properties, while those burned to black ash having warming properties. Similarities in the use and processing of animal medicines across different cultures and nations suggest a universal ethnomedicinal wisdom [28, 65]. These methods likely evolved from long-term practice by the Gelao community and possess certain scientific and rational bases.

The efficacy of animal-derived medicines is broad, capable of treating a wide range of internal and external diseases. Most of these medicines are non-toxic or low-toxic, with high safety margins, rarely causing side effects or toxic reactions, and many also have nutritional value, meeting the Gelao’s nutritional and health needs. The cultural significance of these medicines is substantial, reflecting the Gelao’s traditional knowledge, beliefs, and values. Some medicines are used not only for healing but also in rituals like sacrifices, weddings, funerals, and festivals, manifesting the Gelao’s reverence and gratitude toward animals and their pursuit of harmony and balance with nature and society. In summary, animal-derived medicines used by the Gelao are an important part of their traditional medicine, a precious heritage of their culture, and a significant resource in the global medicinal repository. We should strengthen the protection, inheritance, and development of animal-derived medicines used by the Gelao to better serve human health and well-being.

### The utilization patterns and conservation measures of animal-derived medicines in China

The utilization patterns of medicinal animals vary in different regions and cultures, depending on the availability, accessibility, affordability, acceptability, and efficacy of animal products, as well as the local beliefs, traditions, preferences, and needs of the users. However, some commonalities and general trends have been identified, such as the dominance of mammals and reptiles, the use of various parts and products, and a preference for rare and endangered species. This utilization pattern reflects a deeply rooted tradition that is intertwined with biodiversity conservation challenges, especially regarding endangered species.

The increased demand for animal-derived medicines in TCM has placed additional pressure on wild animal populations. The illegal trade of these products exacerbates the risk of extinction for species that are already vulnerable due to habitat loss, climate change, and other anthropogenic factors. Recognizing the dark side of traditional medicine is crucial for promoting sustainable and ethically responsible practices that respect the rich heritage of traditional medicine while protecting the Earth’s biodiversity. To address the contradiction between resource scarcity and market demand, China has implemented various national strategies and legislative measures aimed at curbing illegal wildlife trade and promoting the conservation of endangered species. This includes compliance with international conventions such as the Convention on International Trade in Endangered Species of Wild Fauna and Flora (CITES), which regulates the trade of endangered wild fauna and flora species to ensure their survival is not threatened. In 2016, the People’s Republic of China enacted the Traditional Chinese Medicine Law, the first law to systematically embody the characteristics of traditional Chinese medicine. It explicitly protects medicinal wild fauna and flora resources, implements dynamic monitoring and regular surveys of medicinal wild fauna and flora resources, establishes germplasm gene banks for medicinal wild fauna and flora resources, encourages the development of artificial cultivation and breeding, and supports the legal protection, breeding, and related research of precious and endangered medicinal wild fauna and flora. For valuable medicinal materials such as *Moschus* (Linnæus, 1758), *Calculus bovis* (C. bovis), Fel Ursi (Bear Bile), and Saiga tatarica (Linnaeus, 1766), which have not been banned, artificial breeding of animals is encouraged as an alternative to hunting wild animals, enhancing the research and promotion of artificial domestication and breeding to transition from wild to domestic. Artificial breeding farms for deer, musk deer, bears, toads, scorpions, centipedes, turtles, and snakes have been successfully established, aiming to achieve a virtuous cycle of resource utilization and environmental protection. The domestication and breeding of medicinal animals is a primary means of ensuring the supply of animal medicines, effectively solving the problem of a lack of available medicines in traditional Chinese medicine clinics due to the depletion of wild resources and the unsuccessful breeding of certain species [66, 67].

### Awareness of wildlife conservation among the Gelao community

The Gelao community, through their long history of production and life, have accumulated rich experience and knowledge in the use of animal-derived medicines. This knowledge reflects not only their understanding and utilization of nature but also their respect and protection of wildlife resources.

Firstly, the Gelao community’s awareness of wildlife conservation stems from their ethnic religious beliefs and cultural traditions. They worship ancestors, revere deities such as Bamboo King, Barbarian King Ancestor, and Mountain God, and believe in animism, viewing animals as incarnations or messengers of spirits deserving reverence and respect. They also have animal-related myths and folktales like ‘The Legend of Yelang King,’ ‘The Tiger-Slaying Hero,’ and ‘Ox Tendon Dance,’ which not only show their close relationship with animals but also reflect their reverence and gratitude toward them [61]. Their ethnic festivals and customs, such as ‘March 3rd Festival,’ ‘Mountain God Sacrifice,’ and ‘Field Mother Sacrifice,’ are closely linked to animals. During these occasions, the Gelao offer sacrifices to animals, seeking their blessings and protection, and expressing gratitude and well-wishes. These religious beliefs and cultural traditions provide a profound spiritual foundation and cultural support for their awareness of wildlife conservation.

Secondly, the awareness of the Gelao community toward wildlife conservation is evident in their use and transmission of ethnic medicine. With a long history and rich knowledge of ethnic medicine, animal-derived medicines form an essential part of Gelao medicine. The Gelao utilize various animal parts, such as fur, bones, organs, blood, and eggshells, to create various medicines for treating diseases and health preservation. In their use of animal-derived medicines, they have developed conservation knowledge and practices, such as refraining from hunting animals during breeding and nursing periods, not killing immature animals, avoiding the use of rare and endangered animal medicines, and preferring domestic animals or purchasing animal medicines from other regions. The Gelao also focus on the transmission and innovation of animal-derived medicines, training ethnic doctors who understand the theories and methods of Gelao medicine, and continuously improving and developing it according to the changes of the times and social needs, thereby better serving the health of the people. These practices of the Gelao not only represent rational utilization of animal-derived medicines but also effective conservation of wildlife resources [66, 68].

Finally, the Gelao’s awareness of wildlife conservation is reflected in their progressive environmental concepts and actions. With societal development and ecological changes, the Gelao have increasingly recognized the importance and urgency of wildlife resources. They actively respond to national wildlife conservation policies and laws, voluntarily abandoning or reducing traditional practices harmful to wildlife conservation, such as hunting, consumption, and trading. They also participate in and support public welfare projects and activities conducive to wildlife conservation, such as establishing nature reserves, promoting ecological tourism, conducting public education, and engaging in volunteer services. The Gelao have established good cooperation and communication with other ethnic groups and social organizations to jointly explore and address the challenges of wildlife conservation, promoting its progress and development. These concepts and actions of the Gelao reflect their active participation in and contribution to wildlife conservation.

The awareness of the Gelao community toward wildlife conservation is an integral part of their ethnic culture and medicine, as well as a significant manifestation of their progressive environmental philosophy and actions. Their awareness and actions in wildlife conservation not only contribute to maintaining and restoring the balance and stability of ecosystems but also help protect and pass on the unique value and charm of their ethnic culture and medicine.

### Current status of traditional knowledge in the use of animal-derived medicines by the Gelao community

The inheritance and dissemination of the Gelao community’s traditional knowledge in using animal-derived medicines face significant risks, a challenge common to most ethnic traditional medicines [69]. Regarding the Gelao’s traditional use of animal-derived medicines, several factors limit their development and continuity. First, the traditional knowledge of the Gelao in using animal-derived medicines is challenged by modern medicine. With the popularization and development of modern medicine, the living and medical conditions of the Gelao have significantly improved. Many common diseases and symptoms can now be effectively treated and alleviated with modern medical methods, reducing reliance on traditional knowledge of animal-derived medicines. Moreover, modern medicine continuously seeks and develops alternatives to animal-derived medicines, like artificially synthesized musk and bear bile acid [70], which are not only effective but also more affordable and readily available, gaining widespread acceptance and use. These challenges from modern medicine significantly diminish the need and value of the Gelao’s traditional knowledge in using animal-derived medicines, sometimes even leading to its dismissal as backward, superstitious, or cruel, resulting in a loss of motivation and confidence in their traditional practices.

Secondly, the transmission and innovation of the Gelao’s traditional knowledge in using animal-derived medicines face difficulties and challenges. This knowledge is mainly transmitted orally and through practice, from older generations of ethnic doctors or herbalists to younger apprentices or successors. This method of transmission requires time and effort and is susceptible to external factors such as population movement, cultural conflicts, and ecological changes, making it difficult to ensure efficiency and quality. Additionally, due to a lack of scientific theory and methodology, the Gelao face challenges in researching and analyzing the components, effects, pharmacology, and side effects of animal-derived medicines, as well as optimizing their preparation, application, combination, and dosage. It is also difficult to explore and discover new functions and indications for these medicines. Thus, the traditional knowledge of the Gelao in using animal-derived medicines often remains at the level of experience and observation, lacking scientific and innovative aspects to meet societal changes and needs.

The current status of the Gelao community’s traditional knowledge in using animal-derived medicines is not optimistic. It faces restrictions and challenges from laws and regulations [71], modern medicine, and difficulties in transmission and innovation, risking its loss and extinction. This situation not only undermines the protection and development of the Gelao’s ethnic medicine but also the diversity and richness of China’s ethnic culture. Therefore, we should pay close attention to the protection and development of the Gelao’s traditional knowledge in using animal-derived medicines. Effective measures should be taken, such as strengthening the formulation and implementation of laws and regulations, supporting and encouraging research and development of artificial breeding and substitutes, and enhancing the documentation and organization of the Gelao’s traditional knowledge in using animal-derived medicines, as well as its transmission.

### Creative applications of exotic medicines in Gelao medicine

Compared to other ethnic groups, the traditional wisdom of the Gelao community in the use of animal-derived medicines is distinct. For instance, the Gelao use silkworm pupae to treat pediatric indigestion, while the Tujia people primarily use them for dietary therapy [72]. The Gelao treat hemorrhoids with toads, while toad skin, known for its tonic properties, is commonly used to treat malignant tumors [73]. The Gelao use Blister Beetles to treat hemangiomas, different from the Bouyei people who use them for carbuncles [74]. The Tujia use them for liver and stomach pain, stomachache, and pediatric indigestion; the Miao use them for chest congestion. The Gelao use mole crickets to treat stone diseases, while the Miao use them for swelling, detoxification, and diuresis [75], the Bouyei for dog bites [76], and the Shui for edema [77]. The Gelao use *Zaocys dhumnades* to treat tremors, while Mongolian medicine uses it for scabies, vision loss, and amenorrhea [78]. The Gelao treat bone fracture pain with leeches, while the Yi apply them topically for drug absorption [79], and the Zhuang use them for amenorrhea, liver cirrhosis, and injuries [80]. The Gelao use snails for umbilical hernias and cramps, the Miao for mumps, the Bouyei for constipation, and the Shui combine them with borneol to treat prolapse [81]. The different ethnic groups exhibit distinct wisdom in the use of animal-derived medicines, varying in the primary diseases treated and the methods of application.

Additionally, the Gelao apply the juice of Blister Beetles to treat hemangiomas; consume roasted sparrows, sans feathers and viscera, to treat chronic cough in the elderly; combine honey bees with honey/middle portion of boy’s urine for mouth and tongue sores/hemoptysis; use tadpoles mixed with borneol in water for mumps; combine *Zaocys dhumnades* with aconite in fumigation therapy for tremors; mix human fingernails with crushed sour jujube seeds for embedded needles; apply a mixture of centipede powder and pig bile to treat stroke-induced facial paralysis; apply swallows’ nest mud mixed with sour soup for testicular pain; and consume ash of burned pig teeth for pediatric epilepsy and snake bites. These unique methods of using medicines are an essential part of Gelao medicinal culture and play a significant role in the everyday health of the Gelao community.

### Defects of traditional animal-derived medicine

Despite the long history and application of traditional animal medicines across various cultures, their use is not without significant drawbacks. First and foremost, the efficacy of certain animal medicines has not been fully validated by modern scientific research. Moreover, the use of wild animals as medicinal resources can pose risks of zoonotic diseases, especially in the absence of appropriate processing and quality control measures [82]. While the use of animal-based Chinese medicinal materials is grounded in traditional experience, it is not always backed by modern scientific evidence [83]. On the other hand, the utilization of wild animals increases the risk of transmitting zoonotic diseases. Although wildlife protection laws have established systems for the artificial breeding and cultivation of wild animals, the potential disease transmission risks associated with other uncontrolled wild animals remain [84]. Consequently, while traditional animal medicines hold an important place in Chinese medicine, there is a pressing need for more scientific research to validate their efficacy. Additionally, appropriate measures must be taken to reduce reliance on wild animal resources, thereby lowering the risk of zoonotic diseases. This approach not only ensures the safety and efficacy of traditional medicines but also contributes to the protection of biodiversity and public health.

## Conclusion

The Gelao community possess an unique insights in the use of animal-derived medicines. We have compiled traditional knowledge of 55 such medicines, identifying several distinct medicinal materials and methods characteristic of the Gelao. This information can provide valuable insights for modern pharmacological research on these medicines. However, this traditional knowledge faces significant risks of discontinuation. Our work not only extends the life of this traditional knowledge but also provides primary data for in-depth research and development of these ethnic medicines. Furthermore, we recommend sustainable utilization and development of animal medicinal resources that can be obtained through domestication.

## Data Availability

All data, materials, and information are collected from the study sites.

## Abbreviations

HUF: The Utilization Frequency
NCSI: National Cultural Significance Index
FQI: Frequency of quotation index
AI: Availability index
FUI: Frequency of utilization index
PUI: Parts used index
MFI: Multifunctional use index
NVI: Nutritional value index
DSI: Drug safety index
